# Functional segregation of rostral and caudal hippocampus in associative memory

**DOI:** 10.3389/fnhum.2025.1509163

**Published:** 2025-02-10

**Authors:** Alicia Nunez Vorobiova, Matteo Feurra, Enea Francesco Pavone, Lennart Stieglitz, Lukas Imbach, Victoria Moiseeva, Johannes Sarnthein, Tommaso Fedele

**Affiliations:** ^1^Department of Psychology, National Research University Higher School of Economics, Moscow, Russia; ^2^Centre for Cognition and Decision Making, Institute for Cognitive Neuroscience, HSE University, Moscow, Russia; ^3^Braintrends Ltd., Rome, Italy; ^4^University Hospital Zurich, University of Zurich, Zurich, Switzerland; ^5^Swiss Epilepsy Center, Zurich, Switzerland; ^6^Children's Hospital, University of Zurich, Zurich, Switzerland

**Keywords:** hippocampus, memory, epilepsy, stereo-EEG, associative memory

## Abstract

**Introduction:**

The hippocampus plays a crucial role in episodic memory. Given its complexity, the hippocampus participates in multiple aspects of higher cognitive functions, among which are semantics-based encoding and retrieval. However, the “where,” “when” and “how” of distinct aspects of memory processing in the hippocampus are still under debate.

**Methods:**

Here, we employed a visual associative memory task that involved encoding three levels of subjective congruence to delineate the differential involvement of the rostral and caudal portions (also referred as anterior/posterior portions) of the human hippocampus during memory encoding, recognition and associative recall.

**Results:**

Through stereo-EEG recordings in epilepsy patients we show that associative memory is reflected by rostral hippocampal activity during encoding, and caudal hippocampal activity during retrieval. In contrast, recognition memory encoding selectively activates the rostral hippocampus. The temporal dynamics of memory processing are manifested by gamma power increase, which partially overlaps with low-frequency power decrease during encoding and retrieval. Congruence levels modulate low-frequency activity prominently in the caudal hippocampus.

**Discussion:**

These findings highlight an anatomical segregation in the hippocampus in accordance with the contributions of its partitions to associative and recognition memory.

## 1 Introduction

Understanding how different cognitive functions are mapped in the hippocampus in its rostrocaudal (or anterior/posterior) dimension is of high relevance both for fundamental research and clinical practice. Earlier studies indicated that the caudal hippocampus (HC) or its’ homologs in non-human animals is mainly involved in spatial memory and navigation (Hartley et al., 2003; Nadel et al., 2013; Poppenk et al., 2013) memory, whereas the rostral HC is associated to the emotional and stress-related response (de Kloet et al., 2018; Dorey et al., 2011; Strange et al., 2014). However, since most of this knowledge on the functional segregation of HC was derived by animal models, it might not be directly applied to the functional organization of the human hippocampus (Murray et al., 2018). Therefore, it is essential to consider physiological differences in memory organization in human and non-human animals, as well as evolutionary diversity. Specifically, the anatomical boundary between rostral and caudal hippocampus in monkeys is less discrete than between the homologous ventral and dorsal HC in rodents (Strange et al., 2014), supported by connectivity studies of the hippocampus showing a non-dichotomous but rather continuous topographical organization (Kjelstrup et al., 2008; Lee et al., 2015; Thompson et al., 2008).

The differential involvement of the rostral and caudal HC across in the context of episodic memory has been addressed also in human studies. Early positron emission tomography (PET) studies described the distinct involvement of the rostral and caudal HC in encoding and retrieval processes for both verbal and non-verbal stimuli, respectively (Lepage et al., 1998). Converging evidence from meta-analyses and functional connectivity data (Dalton et al., 2022; Grady, 2020) suggests heterogeneous involvement of the HC in memory functions, with encoding and retrieval following a rostrocaudal mapping pattern (Fritch et al., 2020, 2021). However, a recent fMRI study has shown that both rostral and caudal HC activity is associated with spatial memory encoding challenging previously consolidated models associating encoding to the rostral HC and that the caudal HC to spatial memory (Sullivan et al., 2024).

Another important variable mediating associative memory is its congruence. When information is congruent with existing knowledge, it facilitates the formation of stronger associations between new and previously stored information (Kahana, 2012; Tse et al., 2007; van Kesteren et al., 2012). This enhances the ability to recall related items and strengthens the overall memory network (Alejandro et al., 2021; Packard et al., 2020), contributing significantly to the effectiveness of associative memory processes (Toyota, 1996). Semantic knowledge, represented by various structures such as schemas, patterns, images, typical situational scenarios, and others (Ghosh and Gilboa, 2014; Gilboa and Marlatte, 2017), is formed through the generalization of multiple episodes. On the other hand, episodic memory, as a constructive process, relies on semantic structures that serve as support for encoding information, consolidation, and intentional recall (Anderson, 1994; Bartlett, 1933; Fernández and Morris, 2018; Piaget, 2003). According to the theory proposed by van Kesteren and collaborators (2010) the congruence of incoming information with previously formed semantic knowledge facilitates its processing, leading to more effective memorization, consolidation, and retrieval of information (Frank et al., 2018; van Kesteren et al., 2012). In turn, reactivation can change the neural correlates of episodic memories, making the memories themselves more schematic and stereotypical (van der Linden et al., 2017; Nadel et al., 2007). Thus, the semantic and episodic memory systems are in a constant reciprocal dynamic interaction.

Here, we hypothesize that the rostral and caudal portions of the human HC are concurrently but differentially involved in memory encoding, recognition, and associative retrieval. Moreover, we hypothesize that congruence-mediated memorization is reflected in the hippocampus activity. To test these hypotheses, we need to track the neural activity at a fine temporal scale along distinct phases of memory processing.

While neuroimaging can anatomically map activation patterns, only intracranial neurophysiological recordings can capture the modulation of local oscillatory neuronal activity (Buzsáki et al., 2012; Johnson and Knight, 2015; Parvizi and Kastner, 2018; Youngerman et al., 2019). Although the oscillatory activity of the HC as a whole has been extensively investigated in the domain of episodic memory (Axmacher et al., 2010; Fell et al., 2001, 2003; Griffiths et al., 2019; Henin et al., 2019; Lega et al., 2012, 2016; Marks et al., 2021; Nahum et al., 2011; Norman et al., 2019; Sederberg et al., 2006, 2007; Staresina et al., 2012, 2013, 2016), the specific contributions of the rostral and caudal HC to recognition and associative memory have not yet been fully elucidated.

Here, we tested our hypothesis by analyzing the modulation of oscillatory power in hippocampal stereo-EEG recordings from epilepsy patients during a long-term associative memory task involving associations of varying congruence. We demonstrated that memory encoding, recognition, and associative retrieval are differentially mediated by the rostral and caudal HC, each characterized by distinct patterns of spectral power modulation.

## 2 Materials and methods

### 2.1 Participants

Eight patients with drug-resistant epilepsy implanted with depth electrodes in the hippocampus for presurgical evaluation of stereo-EEG participated in the study. Two patients were excluded due to the presence of persistent interictal spikes in the recordings. From the remaining patients, two retrieval sessions were excluded from the analysis due to the presence of interictal epileptic discharges and artifacts in the 100% of each recorded session. In total, the analysis included 6 patients for the encoding session (mean age = 30.5, S.E. = 5.6, 5 females) and four patients (mean age = 32, S.E. = 8.6, 3 females) for the retrieval session. All patients were right-handed, had normal vision and provided written informed consent as approved by the institutional ethics review board (PB 2016–02055). The sample size is in line with the sample sizes of other stereo-EEG studies with similar long-term memory paradigms (see Axmacher et al., 2010; Castelhano et al., 2022; Mormann et al., 2007; Staresina et al., 2012; Sweeney-Reed et al., 2016; Vila-Vidal et al., 2023).

### 2.2 Task design

Each participant attended two sessions: encoding and retrieval over two consecutive days. During the encoding session, participants performed an associative task which included an estimation of congruence of encoded material (object-scene pairs). On the next day, during the retrieval session, participants performed recognition and associative memory tests. The mean intersession gap was 23 ± 1 h.

The associative memory task was adapted from van Kesteren et al. (2013). The set consisted of 185 pairs of pictures (one representing an object and one representing a scene). Pictures were recognizable colored photographs representing indoor and outdoor scenes and objects of different sizes. Each object-scene associative pair was unique (i.e., every picture was presented only once to each participant). The pairs were initially constructed to be very congruent (e.g., “book” and “library,” 10% of pairs), of medium congruence (e.g., “earplugs” and “living room,” 80% of pairs) or very incongruent (e.g., “lab” and “beachball,” 10% of pairs). On the first day, participants were presented with a set of 185 object-scene pairs. They received instructions to remember “everything they see on the screen.” Following the presentation of an object-scene pair, the participants estimated the semantic congruence of the object-scene pair using a visual analogue scale ranging from “does not fit” to “fits very well.” Their answers were referred to the congruence of the stimuli.

**Figure 1:**
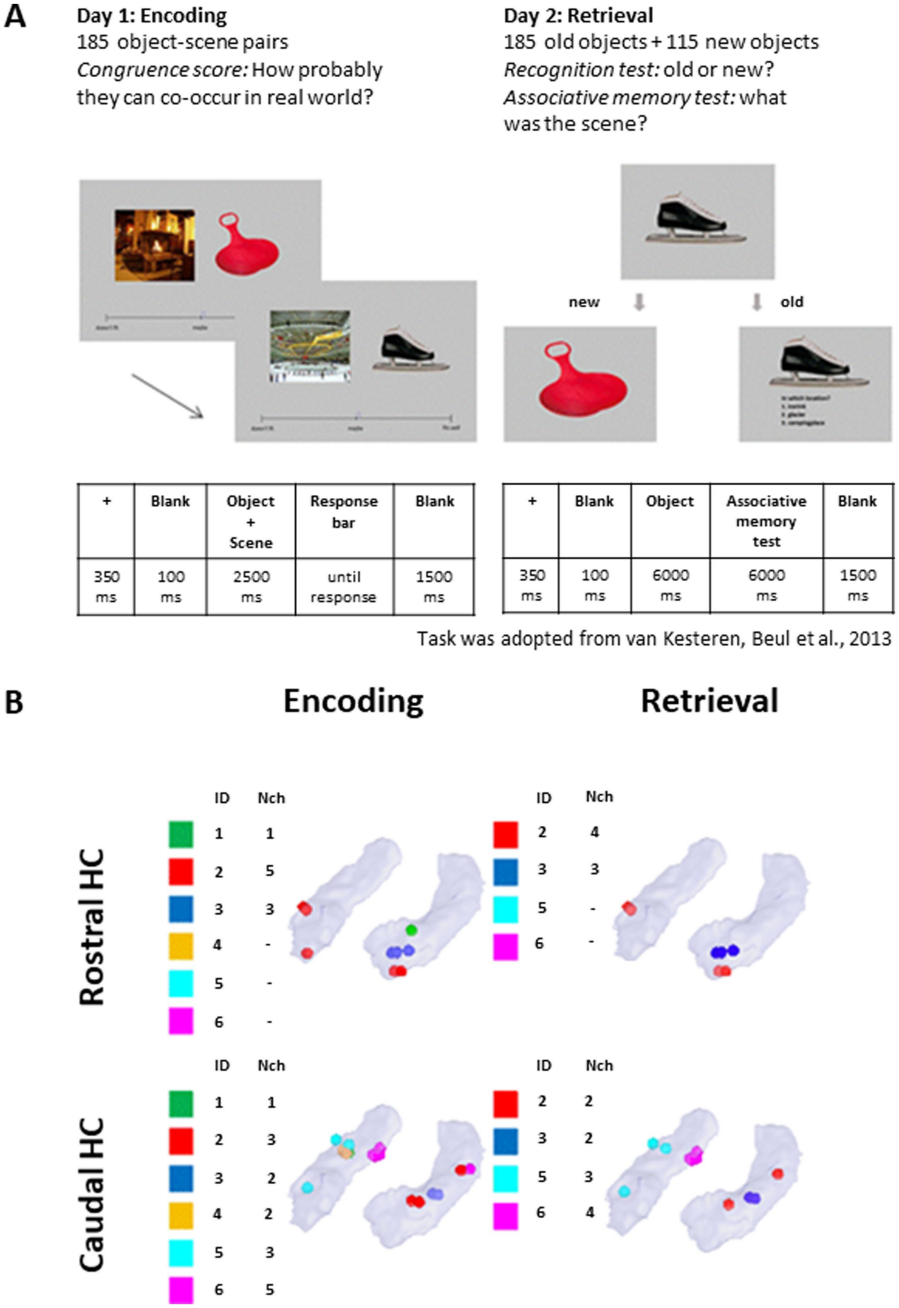
Task description and distribution of electrode contacts. **(A)** Timeline of the behavioral task in the encoding and retrieval sessions. The task was adopted from van Kesteren et al. (2013). **(B)** Distribution of the electrode contacts across the patients’ rostral and caudal HC. ID: Patient; Nch: number of channels. One of the contacts, despite appearing rostral in visualization, is caudal based on native coordinates. MNI coordinates are available in Supplementary Table 1.

The trial structure is shown in Figure 1A. Before stimulus onset a black fixation cross on a gray background was presented for 350 ms, followed by a gray blank screen for 100 ms. The visual stimulus, composed by a pair of pictures representing an object and a scene, was presented for 2.5 s. The object was presented on the right and the scene was presented on the left of the screen. After 100 ms of blank screen, a visual analogue horizontal scale appeared. Participants had unlimited time to answer the question “how well does this object fit this scene?” moving a pointer along the visual analogue scale with a computer mouse. After the congruence rating, the screen remained blank for 1.5 s before the start of the next trial.

On the second day, participants completed the retrieval session, where they were presented with 300 objects (185 old +115 new) and asked to distinguish between familiar items learned in the encoding part and new items (item recognition test). The old and new items were presented in random order. Following the answer “new,” the next item was presented. Following the answer “old,” the participant was asked to remember in which context this item appeared previously and to choose from three options (associative memory test). Options were presented as one or two-word verbal descriptions to achieve more categorical-type recognition than perceptual features recognition (van Kesteren et al., 2013). Finally, the participants were asked how sure they were about their response (“guess,” “not sure,” “very sure”). Participants had 6 s to answer each question. The experiment timeline is shown in Figure 1A.

Multiple-choice options for the associative memory test were manually predefined so that, together with the correct answer, they included two congruent options and one incongruent option (van Kesteren et al., 2013).

The paradigm was implemented in E-Prime (version 2.0.10.147, Psychology Software Tools, Pittsburgh, PA).

### 2.3 Stereo-EEG acquisition

The recording was carried out in the intensive monitoring unit of the Swiss Epilepsy Center in Zürich using electrodes with 8 recording contacts (diameter 1.3 mm, contact length 1.6 mm AD-Tech, www.adtechmedical.com). Stereo-EEG was recorded with an ATLAS recording system (sampling rate 4 kHz, 0.5–1,000 Hz bandpass, Neuralynx, www.neuralynx.com).

Localization and anatomical labeling of contacts was performed using the protocol described in Stolk et al. (2018) based on the Brainnetome Atlas (Fan et al., 2016) after merging the post-operative MR with post-operative CT images in MNI space. The neurosurgeon (LS) confirmed the anatomical labels of the contacts in the participants’ native space (iPlan Stereotaxy 3.0, Brainlab, Germany). Contacts located in the HC and their distribution across patients are shown in Figure 1B.

### 2.4 Pre-processing

The stereo-EEG signal was downsampled to 200 Hz. We adopted Common Average Reference in agreement with previous studies on stereoEEG data (Arnulfo et al., 2015; Mercier et al., 2022). The data was visually inspected in order to remove episodes that included epileptic spikes and technical artifacts. After preprocessing, trial rejection rate was equal to 26% (SEM = 8%) for the encoding data and 28% (SEM = 9%) for the retrieval data across participants. While the noise level may vary across two days of recordings, we compared the percentage of rejected trials across sessions and found no significant difference [*t*(7.04) = 0.12, *p* = 0.91]. Data was then epoched from −2 to 3 s around the stimulus onset. Pre-processing of the stereo-EEG signal was performed in Brainstorm (Tadel et al., 2011).

### 2.5 Experimental design and statistical analyses

#### 2.5.1 Behavioral data

During the encoding phase, each object-scene pair was rated by subjects on a congruence scale ranging from “incongruent” (0) to “congruent” (100). Item pairs were considered incongruent if rated between 0 and 33, intermediate from 34 to 66, and congruent from 67 to 100. To check for significant difference in the total amount of items that got in each congruence bin according to the subjects’ rating, we used the Friedman rank sum test, with the number of items as a dependent variable and congruence as a factor (“Congruent,” “Intermediate,” “Incongruent”). This non-parametric test allows for the comparison of repeated measures across multiple conditions, making it an appropriate choice for our study design with a small sample size.

**Figure 2:**
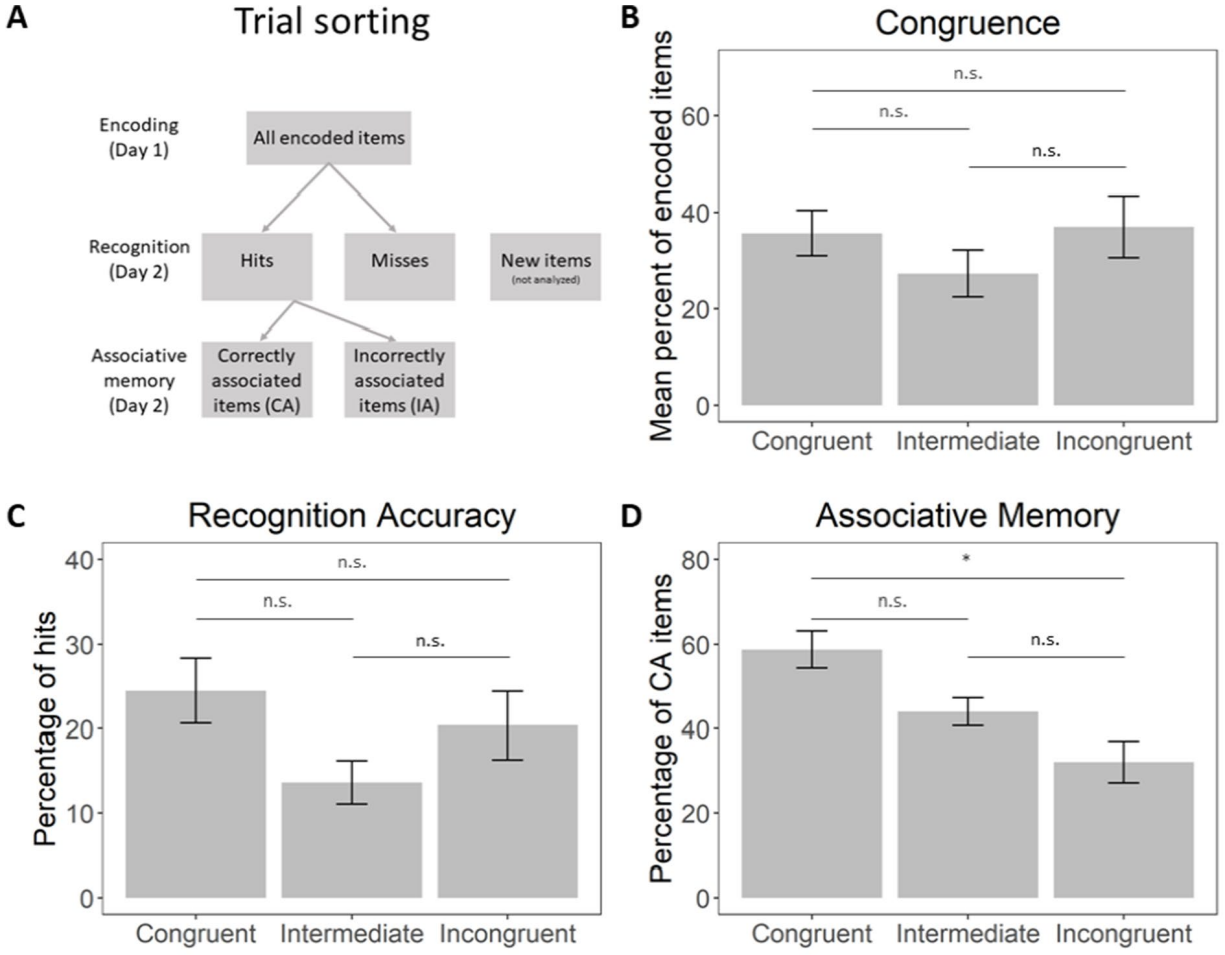
**(A)** A scheme of memory-based trial sorting. **(B)** Percentage of object-scene pairs endorsed as congruent, intermediately congruent, incongruent. **(C)** Percentage of hits within each subjective congruence bin, relative to the total amount of items. **(D)** Percentage correct scene-object associative memory, relative to the total amount of hits. Error bars represent the standard error of the mean (SEM). * - The mean difference is significant at the 0.05 level, n.s. - the mean difference is not significant. Horizontal bars represent the results of Bonferroni corrected *post hoc* Wilcoxon signed-rank tests between congruence levels.

Then, according to the participants’ memory performance, the trials for encoding and retrieval were sorted into four categories (see Figure 2A):

Hits: object items correctly recognized at retrieval as “old” regardless of whether they were associated with the correct scene item;

Misses: object items incorrectly marked as “new” items at retrieval;

Hit and correctly associated (CA): object items correctly recognized as “old” and associated with the correct scene items at retrieval;

Hit and incorrectly associated (IA): object items correctly recognized as “old” but not associated with the correct scene items at retrieval.

For the encoding session analysis, Subsequent Memory Effect (SME) for recognition and associative memory was calculated. Furthermore, for each participant we quantified item recognition performance in terms of discrimination index (DI), defined as the proportion of correctly recognized old items (Hits) among all old items minus the proportion of false alarms among all new items (Snodgrass and Corwin, 1988). We quantified associative memory performance as the proportion of correctly associated items (CA) among all Hits. We tested the hypothesis that participants’ item recognition and associative memory performance were above chance level by a one-sample t-test (against 0 and 0.33, respectively). A Friedman rank sum test with congruence as a factor (“Congruent,” “Intermediate,” “Incongruent”) was used to assess whether memory performance (item recognition and associative memory) differed between items of each congruence level. For post-hoc comparisons, we employed the Wilcoxon signed-rank test with Bonferroni correction to control for multiple comparisons. The significance level for all tests was established at *p* < 0.05.

#### 2.5.2 Neurophysiological data

The analysis of stereo-EEG data was performed separately for rostral and caudal HC with respect to item recognition and associative memory. Baseline corrected epochs (−500 to −100 ms baseline window) underwent a time-frequency analysis with the following parametrization for low- and high-frequency ranges (Staresina et al., 2016): for the frequency range 2–29 Hz we applied a 1 Hz resolution, 5 cycle temporal window and a Hann taper. For the frequency range 30–100 Hz we applied a 5 Hz resolution, 400 ms temporal window and seven orthogonal Slepian tapers (resulting in spectral smoothing with a frequency of approximately ±10 Hz). The resulting power maps were normalized with respect to the prestimulus baseline window (from −1,500 to −500 ms). A 2 s time window (0 to 2 s from the stimulus onset) was selected to capture both early and late hippocampal effects described in previous studies (Barborica et al., 2023; Staresina et al., 2012). We were interested in characterizing the modulatory effect of memory performance and congruence level on the oscillatory power in the rostral and caudal HC during the encoding and the retrieval phase. Since congruence and associative memory accuracy are behaviorally linked (Figure 2B, also see Anderson, 1994; Poppenk et al., 2010), a generalized linear model (GLM) analysis was more appropriate for our study design than several separate tests (Cohen, 2019). We therefore adopted a GLM framework to relate time-frequency power (dependent variable) with the mixed-effect of categorical behavioral conditions (independent variables). Two GLM analyses were implemented trial-wise separately for recognition and associative memory. Recognition memory was subjected to a mixed-effects GLM with the regressors “Recognition memory” and “Congruence” (defined as time-frequency power ~ 1 + a1*Recognition memory + a2*Congruence + a3*Congruence x Recognition memory, with two categorical explanatory variables: “Recognition memory”: “Hits” and “Misses” and “Congruence”: “Congruent,” “Intermediate,” “Incongruent”). For the associative memory, the GLM was defined similarly to the recognition model, but with the “Associative memory” variable having other two levels, “CA” and “IA”.

We computed regression coefficients for each time-frequency point (function fitlm.m, Matlab) and evaluated the linear mixed effects by ANOVA (anova.m, MATLAB). The resulting *F*-values and *p*-values populated the *F*-map and *p*-map, respectively, for each of the considered independent variables. We considered only time-frequency points with *p*-values <0.05. In order to control for multiple comparisons we applied a non-parametric cluster analysis. In each *F*-map, we retained only significant clusters larger than the clusters obtained from the null-distribution (resulted from 500 permutations of shuffled data) with a threshold of *p* = 0.05.

Following the significant effects detected by the GLM model, we directly compared time-frequency maps obtained separately for the rostral and caudal HC during encoding and retrieval for the different trial categories. Cluster analysis with 20,000 permutations was used to determine pairwise statistical significance between CA, IA and Miss, and between different congruence levels (Maris and Oostenveld, 2007). The analysis of preprocessed stereo-EEG data was based on the FieldTrip toolbox (Oostenveld et al., 2011).

## 3 Results

### 3.1 Behavioral results

Firstly, we estimated the distribution of items classified by the subjects as congruent, intermediate, and incongruent (Figure 2B). A Friedman rank sum test with congruence as a factor did not reveal significant differences in their amount: *χ*^2^(2) = 2.33, *p* = 0.311. The Spearman correlations between individual subjective and objective congruence rates are presented in Supplementary Table 2 (mean R^2^ = 0.27, all *p*-values <0.001, showing a significant correlation between subjective and objective rates).

Secondly, based on the memory performance of the participants, the encoding and retrieval trials were classified into four categories (see *Materials and Methods* and Figure 2A): hits, misses, correctly associated items (CA) and incorrectly associated items (IA). Behavioral performance in terms of recognition memory and associative memory in the recorded patients, though lower than in van Kesteren et al. (2013) study, was in line with the expected performance in healthy individuals (Snodgrass and Corwin, 1988). Subjects’ individual performance metrics are presented in Table 1. Recognition memory performance, provided by a discrimination index (DI), was significantly above chance level (0) [*t*(5) = 10.91, *p* < 0.001, *d* = 4.46]. Associative memory performance, measured as a proportion of correctly associated items, was above chance level (0.33) [*t*(5) = 4.63, *p* = 0.006, *d* = 1.89]. Mean (SEM) values of memory performance are shown in Table 1.

**Table 1:** Behavioral performance across item-scene congruence conditions.

Metric	Subject 1	Subject 2	Subject 3	Subject 4	Subject 5	Subject 6	Mean (SEM)
Congruent item-scene pairs	0.55	0.37	0.38	0.20	0.30	0.33	0.36 (0.05)
Intermediate congruent item-scene pairs	0.37	0.19	0.25	0.47	0.19	0.17	0.27 (0.05)
Incongruent item-scene pairs	0.08	0.44	0.37	0.33	0.50	0.50	0.37 (0.06)
Hits	0.71	0.49	0.64	0.41	0.58	0.69	0.59 (0.05)
Correctly rejected items (CR)	0.88	0.90	0.79	0.92	0.94	0.83	0.87 (0.02)
Discrimination index (DI)	0.59	0.39	0.43	0.33	0.52	0.52	0.46 (0.10)
Correctly associated items (CA)	0.54	0.43	0.48	0.38	0.42	0.58	0.46 (0.03)

Finally, we assessed the distribution of correct answers (Hits and CA) in every congruence bin. The percentage of hits at each congruence level is represented in Figure 2C, while the percentage of CA is shown in Figure 2D. A Friedman rank sum test with congruence as a factor did not reveal significant differences between the amount of correctly recognized items of each congruence level [*χ*^2^(2) = 2.33, *p* = 0.311]. However, a Friedman rank sum test with congruence as a factor showed significant differences in the amount of correctly associated items at each congruence level [*χ*^2^(2) = 7.00, *p* = 0.030]. Pairwise Wilcoxon rank-sum tests were conducted to compare the levels of CA across the congruence conditions. The results, adjusted using the Bonferroni correction, revealed a significant difference between the congruent and incongruent conditions (*p* = 0.026). No significant differences were found between the congruent and intermediate conditions (*p* = 0.195) or between the incongruent and intermediate conditions (*p* = 0.195). Therefore, associative memory performance was significantly higher for congruent than incongruent items, indicating that congruent object-scene pairs were more likely to be correctly recalled.

### 3.2 Neurophysiology of recognition memory and associative memory

We quantified the modulation in the time-frequency domain in relation to recognition memory and associative memory performance and subjective congruence estimation. We tested the recognition memory effect through a mixed-effects GLM with oscillatory power as a dependent variable and “Recognition memory” (“Hits” and “Misses”) and “Congruence” (“Congruent,” “Intermediate,” and “Incongruent”) as explanatory categorical variables. We tested the associative memory effect through a mixed-effects GLM with oscillatory power as a dependent variable and “Associative memory” (“CA” and “IA”) and “Congruence” (“Congruent,” “Intermediate,” and “Incongruent”) as explanatory categorical variables. This framework was applied to rostral and caudal HC during the encoding (day 1) and retrieval (day 2) sessions separately.

For a complete overview of the GLM models, direct comparison of trials sorted by memory performance and direct comparison of trials sorted by congruence rating (see Supplementary Figures 1, 2).

#### 3.2.1 The role of rostral HC in recognition memory and associative memory

**Figure 3:**
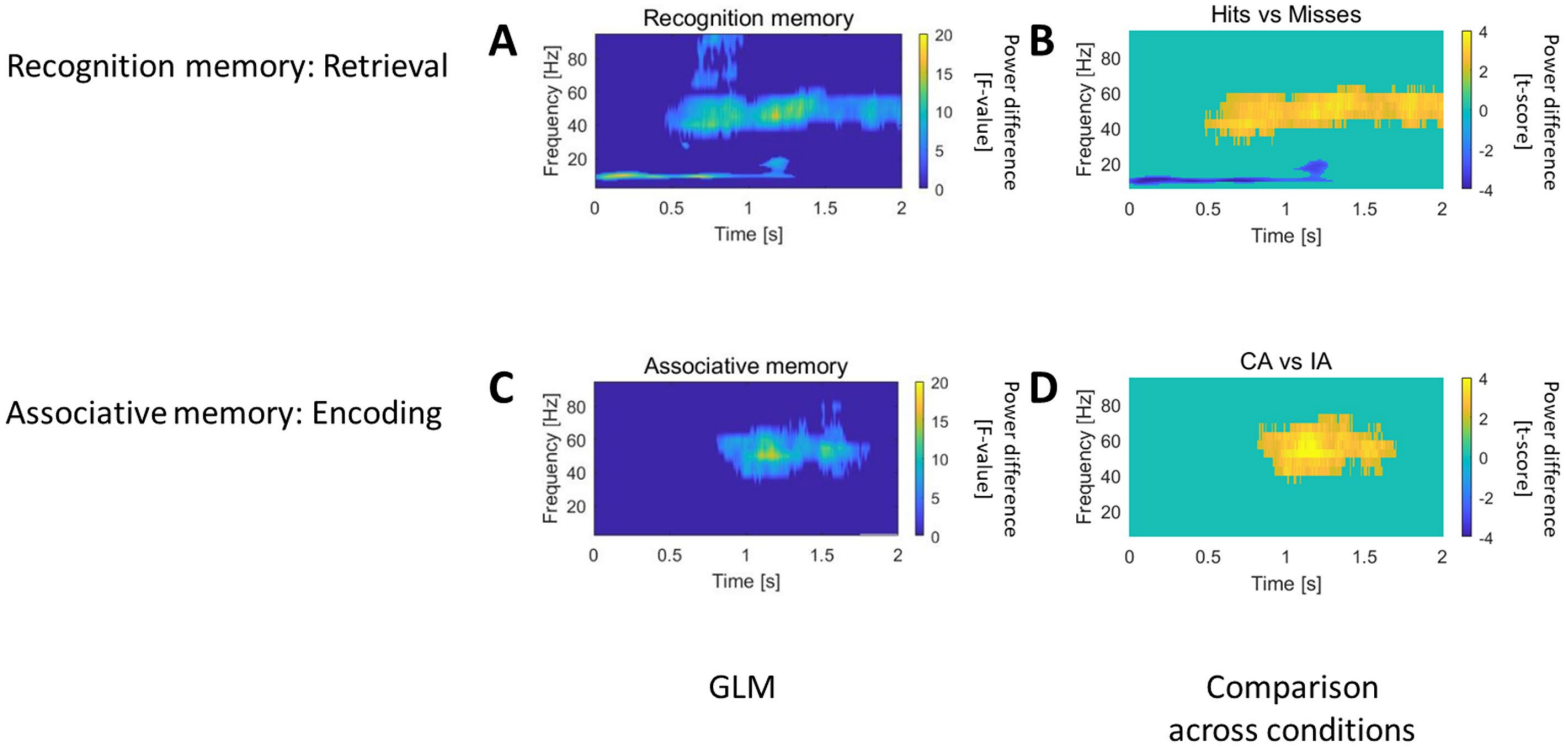
Recognition and Associative memory effects in the rostral HC. **(A)** The recognition memory GLM identified an effect of the “Recognition memory” factor during retrieval. This effect was characterized by early low-frequency power modulation (0–1 s), followed by gamma-band modulation (0.5–2 s). **(B)** Pairwise comparison of Hits and Misses trials revealed that correct item recognition was linked to an early and sustained suppression of oscillatory power in the theta/alpha range (5–13 Hz) lasting up to 1 s. This suppression was succeeded by a sustained gamma power increase (40–60 Hz) starting around 0.5 s and continuing up to 2 s post-stimulus. **(C)** The associative memory GLM outlined an “Associative memory” factor effect during encoding, marked by gamma-band modulation beginning approximately 1 s after stimulus presentation. **(D)** Pairwise comparison of CA and IA trials showed that subsequently correctly associated items were characterized by a gamma power increase (40–70 Hz) starting around 1 s after the object-scene pair presentation. For all panels, t = 0 corresponds to the stimulus onset.

In the rostral HC, the recognition memory GLM revealed an effect for the factor “Recognition memory” during the retrieval session featuring early low-frequency power modulation (0–1 s) followed by gamma band modulation (0.5–2 s, Figure 3A). The direct comparison of Hits and Misses trials demonstrated that correct item recognition was associated with an early-onset sustained suppression of oscillatory power in the low frequency theta/alpha range (5–13 Hz) up to 1 s. This suppression was followed by a sustained increase in gamma power (40–60 Hz) starting around 0.5 s and lasting up to 2 s after stimulus onset (Figure 3B). Importantly, no significant differences were found for CA vs. IA trials (see Supplementary Figure 2F), suggesting that the rostral HC is specifically involved in item recognition but not in associative memory.

In the associative memory GLM, an effect of the factor “Associative memory” was observed during the encoding session, characterized by gamma-band modulation beginning approximately 1 s after stimulus presentation (Figure 3C). A direct comparison of CA and IA trials revealed a gamma power increase (40–70 Hz) for subsequently correctly associated items compared to subsequently incorrectly associated items, emerging around 1 s after the presentation of the object-scene pair (Figure 3D).

Successful recognition in the rostral HC during retrieval was characterized by a decrease in low-frequency power followed by an increase in gamma power. The timing of these oscillatory power modulations suggests that the suppression of low-frequency power may facilitate the recollection of information from cortical structures, while the gamma power increase likely reflects on-site encoding and comparison with the recollected information. The successful subsequent association of object-scene pairs was marked by a distinct increase in gamma power.

#### 3.2.2 The role of caudal HC in recognition memory and associative memory

**Figure 4:**
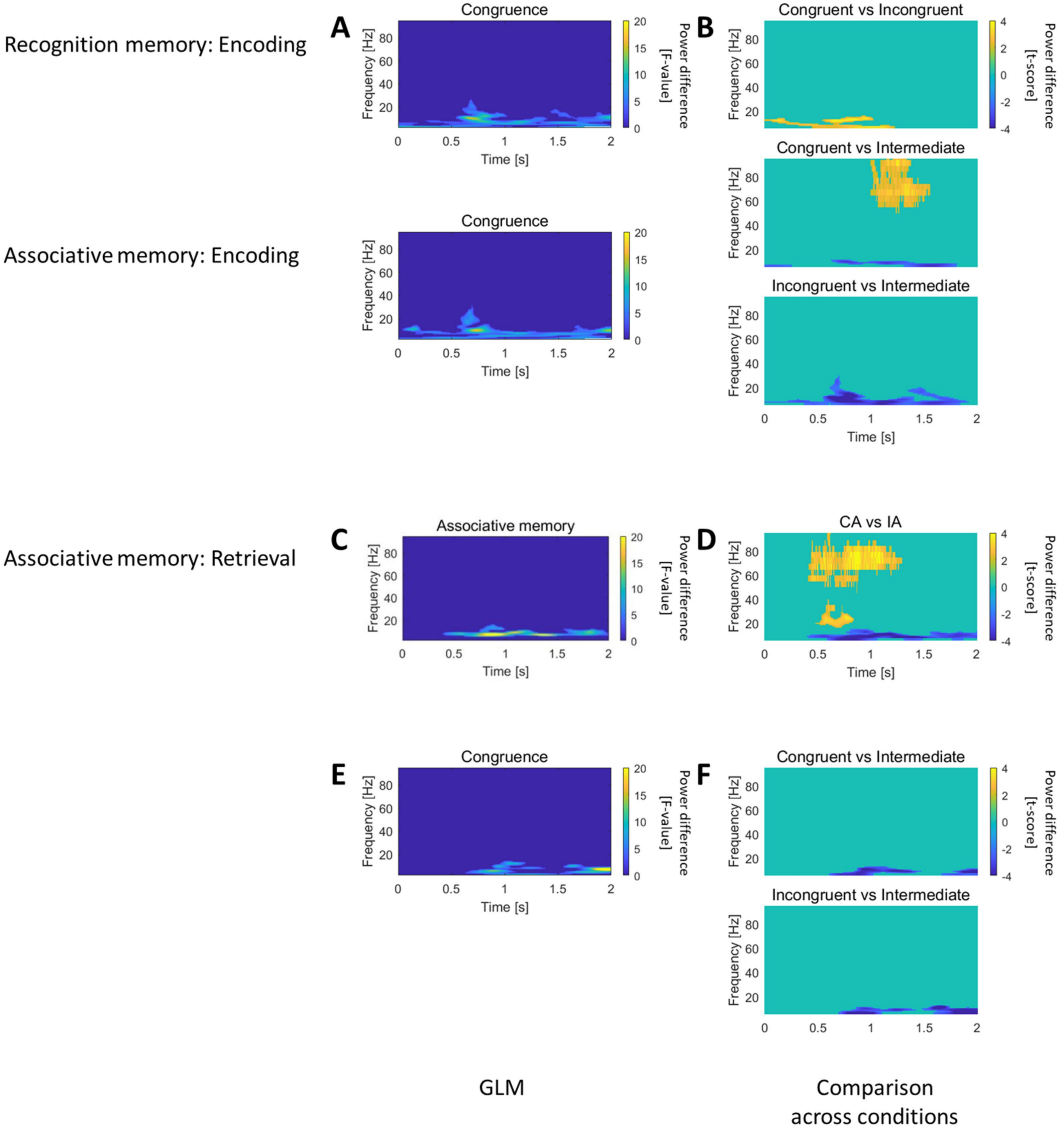
Recognition and Associative memory effects in the caudal HC. **(A)** In the caudal HC, both recognition and associative memory GLM analyses revealed an effect for the “Congruence” factor during encoding, with modulation in the low-frequency spectral range. **(B)** Pairwise comparison of trials with different level of congruence, showed that intermediate congruent trials exhibited higher power, followed by congruent and incongruent trials. **(C)** The associative memory GLM outlined an effect for the “Associative memory” factor during retrieval, characterized by low-frequency power modulation. **(D)** Pairwise comparison of CA vs. IA trials during retrieval demonstrated increased gamma power and decreased theta/alpha power for correctly associated items. **(E)** The associative memory GLM identified an effect for the “Congruence” factor, featuring low-frequency power modulation. **(F)** Pairwise comparisons of trials with different congruence levels showed dominant low-frequency power for intermediate congruent trials.

In the caudal HC, both recognition and associative memory GLM revealed an effect for the factor “Congruence” during the encoding session, with long-lasting modulation in the low frequency spectral range (Figure 4A). The direct comparison across trials of different congruence levels demonstrated higher power for the intermediate congruent trials, followed by congruent and incongruent, as shown in Figure 4B.

The associative memory GLM provided an effect at retrieval for the factor “Associative memory” (Figure 4C) and an effect of the factor “Congruence” (Figure 4E), both featuring low frequency power modulation. The direct comparison between CA and IA at retrieval highlighted increase in gamma and decrease in theta/alpha ranges for correctly associated items (Figure 4D). The direct comparison across trials of different congruence levels revealed a dominant low frequency power for intermediately congruent trials (Figure 4F).

Therefore, the caudal HC contributes to encode congruence information with synchronization/desynchronization in the low frequency domain. Concomitantly, the caudal hippocampus actively participated to retrieve the association between object and scene.

## 4 Discussion

We investigated the functional and anatomical segregation of recognition and associative memory processing along the longitudinal axis of the human HC. Our study tested the hypothesis that recognition and associative memory involve distinct neural activity patterns, which we characterized through time-frequency power modulation analyses. Specifically, we observed that that rostral HC gamma activity during encoding was related to subsequent associative memory performance. During retrieval, correct item recognition was associated with an early low-frequency power decrease followed by a gamma power increase in the rostral HC. Moreover, associative recall at retrieval selectively engaged the caudal HC, showing a distinct pattern of gamma power increase accompanied by a low-frequency power decrease. Additionally, during encoding, we found that congruence levels modulated low-frequency activity in the caudal HC, highlighting its role in processing associative information based on semantic congruence.

### 4.1 Recognition memory

At encoding, our generalized linear model (GLM) analysis did not reveal any significant Subsequent Memory Effect (SME) for either the rostral or caudal portions of the hippocampus (Supplementary Figures 1A,B, top left panels). This finding aligns with established evidence suggesting that hippocampus-independent processes support the encoding of items that are later correctly recognized (Ranganath et al., 2004; Staresina and Davachi, 2006).

During retrieval, however, correct item recognition was specifically associated with an early alpha band power decrease within the rostral HC (Figures 3A,B, top right panel). Recognition involves both perceptual processing of the stimulus and a familiarity-based matching-to-sample search. The observed alpha suppression likely reflects the inhibition of low-frequency idling rhythms, facilitating information exchange between the HC and cortical regions. This is consistent with previous findings that link alpha activity suppression to resource allocation during memory and attention-related tasks (Klimesch, 2012; Klimesch et al., 2007). In this context, the alpha power decrease could represent an adaptive mechanism for engaging strategic memory search (Griffiths et al., 2019; Iemi et al., 2022).

Following the alpha suppression, we observed a subsequent gamma power increase in the rostral HC (Figures 3A,B). Gamma oscillations have been associated with the formation of large-scale cortical memory networks, which temporarily integrate distributed neural representations (Busch et al., 2006; Engel et al., 1990; Jacobs and Kahana, 2009; Martinovic et al., 2007). Interestingly, our findings reveal that this gamma activation occurs approximately 0.5 s after stimulus presentation and follows the alpha suppression phase. Previous research has shown that familiarity-driven cortical gamma activity often precedes hippocampal gamma responses (Supp et al., 2007). In this respect, the gamma power increase observed during retrieval likely reflects not only re-encoding of the stimulus or reactivation of memory traces but may also contribute actively to the computational processes underlying recognition.

### 4.2 Associative memory

During encoding, the rostral HC showed a selective Subsequent Memory Effect (SME), characterized by an increase in gamma power for correctly associated items compared to incorrectly associated items and misses (Figure 3C,D). Gamma power increases during encoding are widely recognized as key mechanisms for item memory formation and episodic binding, facilitating the integration of individual elements into cohesive memory traces (Hanslmayr et al., 2016; Headley and Weinberger, 2011; Henin et al., 2019; Sederberg et al., 2003; Staresina et al., 2016). This process appears to be particularly associated with high-frequency gamma activity, which has been linked to local processing and network coordination during memory encoding (Burke et al., 2014; Griffiths et al., 2019; Jobst and Cascino, 2015; Tort et al., 2009).

At retrieval, successful associative memory recall was accompanied by a distinct pattern of neural activity in the caudal HC, with concurrent decreases in low-frequency power and increases in high gamma power (Figures 4C,D). These findings build upon previous evidence linking hippocampal activity to associative memory retrieval (Griffiths et al., 2019; Staresina et al., 2016), but our results uniquely highlight the specific engagement of the caudal HC during this process. The combined modulation of low- and high-frequency power may reflect a dual mechanism: low-frequency power decreases likely facilitate inter-regional communication by reducing inhibitory rhythms, while gamma power increases are associated with local computations required for memory retrieval.

### 4.3 Congruence

During encoding, caudal hippocampus (HC) activity exhibited a greater decrease in low-frequency power for congruent and incongruent items compared to intermediate ones (Figures 4A,B). Concurrently, a similar pattern was observed during the retrieval of associative memory (Figures 4E,F). This finding highlights the role of the caudal HC in associative encoding and provides new evidence on how congruence modulates hippocampal dynamics. While ripple activity is strongly associated with memory encoding and is thought to be predominantly driven by the caudal HC (Jiang et al., 2019), our results complement this framework by demonstrating that low-frequency power suppression in the caudal HC is also sensitive to the congruence of associative information.

Congruence is a critical factor in memory processing, as it determines the compatibility of new information with pre-existing knowledge structures, such as schemas. According to the theories proposed to explain schema-based processing, congruent information is processed more efficiently because it integrates seamlessly into existing frameworks, while incongruent information requires additional resources for encoding and integration (Ghosh and Gilboa, 2014; van Kesteren et al., 2012). The observed low-frequency power decreases for both congruent and incongruent items suggest that the caudal HC plays a dual role in encoding: facilitating rapid integration of congruent information while also adapting to the demands of encoding incongruent inputs. This dual role underscores the flexibility of the caudal HC in mediating familiarity-driven and novelty-driven processes. A similar pattern is observed during retrieval, reflecting the continuity of underlying neural mechanisms across encoding and retrieval. Strongly congruent and strongly incongruent items are retrieved more easily due to their clear fit or distinct separation from existing schemas. In contrast, items with moderate congruence demand greater cognitive effort as new associations must be established.

### 4.4 Schema-dependent memory and timing

The encoding of associative material is accompanied by an increase in hippocampal (HC) high gamma power (Henin et al., 2019), which may reflect the integration of items into cohesive memory representations. Successful retrieval (recognition and recall), on the other hand, has been associated with the modulation of theta (Anderson et al., 2010; Foster et al., 2012; Sederberg et al., 2006) and gamma rhythms (Sederberg et al., 2007; Steinvorth et al., 2010), and their interaction (Foster et al., 2013; Mormann et al., 2005). Theta rhythms are thought to coordinate the activity of distant brain structures during memory retrieval, while gamma oscillations provide a foundation for localized computations and integration (Knight and Eichenbaum, 2013; Von Stein and Sarnthein, 2000; Watrous et al., 2013).

Our findings contribute to the understanding of the HC’s role in associative memory by providing evidence for distinct spatial and functional activation patterns along the rostrocaudal axis. Initially, the HC was hypothesized to function primarily as a novelty detector (Brodt et al., 2016; Rutishauser et al., 2008; Tulving et al., 1996). More recent evidence has demonstrated its involvement in context recall and feature binding, extending its role beyond novelty detection to include object-context integration (Staresina et al., 2013; Staresina and Davachi, 2006).

In our study, we examined HC activity in object-context associations, which inherently involve episodic experiences and semantic knowledge. These processes align with the concept of “predictable ambiguity,” where stimuli can have multiple meanings depending on contextual variations (Morris, 2006). The HC appears to resolve such ambiguity through a dynamic interaction of gamma rhythms that segregate parallel computational processes. Specifically, at retrieval, we observed distinct patterns of HC gamma activity: higher gamma activity in the caudal HC during associative memory retrieval and lower gamma activity in the rostral HC during item recognition. This functional dissociation supports the hypothesis that gamma oscillations can disentangle interfering computational processes (Colgin et al., 2009) coding separately for novel and familiar information. Presumably, variability in the gamma spectral profile could code specifically for novel and well-known information. Slow gamma oscillations are supposed to be driven by intrinsic HC pacemakers, while fast gamma oscillations are thought to originate from the medial entorhinal cortex (Bragin et al., 1995; Colgin et al., 2009). Interneuronal activity within the HC may mediate the competitive relationship between these states, enabling the flexible encoding and retrieval of associative information (Colgin, 2015; Leão et al., 2012).

To further clarify the spatial and temporal dynamics of the information flow from and toward the HC, future research should incorporate concomitant extrahippocampal sampling. Evidence from fMRI studies has identified two functional subnets within the memory network: an early subnet associated with high-level visual perception and a later one linked to top-down control mechanisms (Buckner and Koutstaal, 1998). However, the detailed time-frequency profile of these subnets remains poorly understood. Current evidence suggests that memory processes involve gamma activity increases in brain regions adjacent to the HC, including the temporal cortices (Fell et al., 2001, 2003; Matsumoto et al., 2013), medial prefrontal cortex (Milivojevic et al., 2015; van Kesteren et al., 2010, 2013), inferior frontal gyrus (Burke et al., 2014; Hoffman et al., 2015; Long et al., 2014), angular gyrus (Davis et al., 2020; Davis and Yee, 2019), precuneus (Brodt et al., 2016) and posterior parietal cortex (Long et al., 2014). Our findings suggest that rostrocaudal HC activity patterns contribute to understanding the mechanisms underlying the associative memory network. By characterizing the differential roles of the rostral and caudal HC, we offer insights into how distinct hippocampal regions coordinate with broader cortical networks to support associative memory retrieval.

### 4.5 Differential roles of rostral and caudal hippocampus

The anterior (rostral) and posterior (caudal) partitions of the human hippocampus (HC) play distinct roles in memory function (Strange et al., 2014). The HERNET model, based on a meta-analysis of fMRI memory studies, suggests that the rostral HC is predominantly associated with the dorsal attentional network, supporting external information processing, while the caudal HC is linked to internal-oriented attention processes mediated by the default mode network (Fritch et al., 2020; Kim, 2015). For example, the two partitions are actively but differentially involved in working memory processing (Grady, 2020), which relies on rostrocaudal information flow (Li et al., 2021). Additionally, the caudal HC has been shown to play a critical role in the recall of verbal information (Lin et al., 2019). Our findings align with the predictions of the HERNET model, demonstrating that successful encoding of episodic information is supported by rostral HC activity, while episodic retrieval relies on caudal HC activity. At retrieval, the caudal HC is specifically involved in recollection rather than familiarity-based item recognition, consistent with the model’s predictions (Kim, 2015). This differentiation suggests that associative recall engages more complex, semantic-based mechanisms that are constructive in nature, whereas visual recognition primarily involves re-encoding and comparative processes.

The functional distinction we observed supports the idea that the rostral HC is optimized for the integration of external sensory inputs during encoding, facilitating episodic memory formation. In contrast, the caudal HC appears to support the retrieval of associative information by reconstructing contextual and semantic details stored across memory networks. This rostrocaudal segregation reflects the broader organizational principles of the HC, where anterior and posterior regions are specialized for distinct attentional and mnemonic functions. Our findings further emphasize the importance of understanding these differential roles to fully characterize how the HC contributes to complex memory processes.

### 4.6 Limitations

The current study provides evidence of the multifaceted involvement of the human HC in memory processing. However, some limitations should be considered. First, the current study has a low sample size (*N* = 6). However, this limitation is typical for stereo-EEG studies since they are strictly constrained by the capacity of surgical centers and high data dropout due to patient-specific clinical factors (Youngerman et al., 2019). Despite the small cohort, comparable sample sizes have been employed in other stereo-EEG studies that have provided critical insights into memory processing (Axmacher et al., 2009, 2010; Castelhano et al., 2022; Mormann et al., 2007; Staresina et al., 2012; Sweeney-Reed et al., 2016; Vila-Vidal et al., 2023). Importantly, consistent behavioral and neural patterns were observed within our cohort, supporting the robustness of our findings despite the sample size limitation. Second, confidence rates were not analyzed because the distribution of responses across different categories was highly uneven. Specifically, certain combinations of congruence and confidence levels were underrepresented (e.g., trials with High congruence but Low confidence accounted for only about 4% of all trials). This limited data in certain categories made it difficult to conduct a meaningful statistical analysis, as the sample sizes were too small to draw reliable conclusions. Besides, due to the low number of high-confidence responses, we cannot completely rule out the possibility that the observed effect is partially influenced by a guessing strategy. However, if the relationship between congruence and associative memory were merely a by-product of guessing (i.e., participants choosing the congruent option when they do not remember well), we would expect accuracy in such case to be below chance level. Finally, the study focused exclusively on the HC, given the spatial constraints of stereo-EEG, which is guided by presurgical clinical hypotheses (Parvizi and Kastner, 2018; Youngerman et al., 2019). While this limited the ability to capture broader hippocampal-cortical interactions, it allowed for a detailed delineation of the functional and anatomical segregation along the rostrocaudal axis of the HC. These findings provide a foundation for future investigations into hippocampal-cortical dynamics and their role in associative memory processing.

While these limitations impose some constraints on the generalizability of the results, the current study contributes critical evidence to the field and highlights the importance of further research to expand our understanding of hippocampal function in associative memory.

### 4.7 Clinical significance

The functional segregation of the hippocampus (HC) along its rostrocaudal axis has important implications for presurgical evaluation and the minimization of cognitive loss. Current evaluations of residual hippocampal functionality primarily rely on tomography studies (Montaz-Rosset et al., 2019; Vinton et al., 2007; Wong et al., 2010) and functional imaging protocols such as fMRI (Schacher et al., 2006), which are applicable to non-implanted patients (Buck and Sidhu, 2020; Duncan et al., 2016). While fMRI and EEG studies have demonstrated correspondence for slower time scales (Fedele et al., 2020; Shamshiri et al., 2019), neural dynamics characterized by fast modulations, such as gamma oscillations, are better captured by neurophysiological recordings like stereo-EEG. In addition, lesions can induce significant functional reorganization within the core memory network (Alessio et al., 2013; Benke et al., 2006; Bernhardt et al., 2019; Golby et al., 2002; Tsukiura et al., 2002). Understanding memory-related patterns of rostrocaudal HC activity can provide critical insights for the interpretation of neuropsychological test outcomes, especially in cases where compensatory mechanisms or reorganization might obscure traditional markers of HC function. By clarifying these memory-related activity patterns, our findings can inform surgical planning and enhance rehabilitation protocols for patients with hippocampal lesions or dysfunction. For example, knowledge of rostrocaudal functional specialization can guide surgeons in preserving critical memory-related regions, reducing the risk of cognitive impairment. Moreover, such insights can help design personalized rehabilitation protocols aimed at leveraging residual HC function or enhancing compensatory mechanisms (de Andrade Morange et al., 2022; Wang et al., 2023).

In summary, the study’s contributions to understanding HC functional segregation have the potential to improve both the diagnostic and therapeutic strategies employed in clinical settings, ensuring better patient outcomes and more targeted interventions.

## 5 Conclusion

Our findings highlight the differential roles of the rostral and caudal hippocampus (HC) in associative memory processing along the longitudinal axis. Specifically, the associative subsequent memory effect during encoding is reflected by rostral HC activity, whereas associative retrieval predominantly engages the caudal HC. This functional segregation suggests that the rostral HC is primarily involved in encoding new associative information and recognizing previously learned items, while the caudal HC supports the retrieval of associative information. Importantly, activity in the rostral part of the HC accompanies the encoding of new associative information and the retrieval of old items, while activity in the caudal HC supports the retrieval of associative information. In both encoding and retrieval phases, the modulation of oscillatory power in the caudal HC precedes that in the rostral HC. This temporal dynamic delineates a system in which the subdivisions of the HC integrate complementary aspects of associative and recognition memory. During congruence estimation, the rostral HC plays a prominent role in associative retrieval, whereas the caudal HC serves as a congruence detector during encoding and modulates memory processing across different levels of congruence.

The timing of oscillatory power changes during retrieval further supports this functional specialization. Suppression of low-frequency power likely facilitates the recollection of information from cortical structures, while the subsequent enhancement in gamma power may represent on-site re-encoding and comparison with recollected information. These findings provide new insights into how the HC orchestrates memory processes, balancing inter-regional communication and localized computations.

In conclusion, this study underscores the functional segregation of the rostral and caudal HC in key memory functions, including encoding, recognition, and associative recall. By delineating the distinct roles and temporal dynamics of HC subdivisions, our findings contribute to a deeper understanding of hippocampal organization and its role in complex memory processes.

## Data Availability

The datasets presented in this article are not readily available because the raw materials are confidential clinical data. Requests to access the datasets should be directed to Alicia Vorobiova, alicianunez.v@gmail.com.

## References

[ref9001] Alejandro R. J. Packard P. A. Steiger T. K. Fuentemilla L. Bunzeck N. (2021). Semantic congruence drives long-term memory and similarly affects neural retrieval dynamics in young and older adults. Front. Aging Neurosci. 13:683908. doi: 10.3389/fnagi.2021.683908 34594212 PMC8477023

[ref1] Alessio A. Pereira F. R. S. Sercheli M. S. Rondina J. M. Ozelo H. B. Bilevicius E.. (2013). Brain plasticity for verbal and visual memories in patients with mesial temporal lobe epilepsy and hippocampal sclerosis: an fMRI study. Hum. Brain Mapp. 34, 186–199. doi: 10.1002/hbm.21432, PMID: 22038783 PMC6870348

[ref2] Anderson R. C. (1994). Theoretical models and processes of reading. Role of readers’ schema in comprehension, learning, and memory. Mahwah, NJ: Erlbaum Publishing.

[ref3] Anderson K. L. Rajagovindan R. Ghacibeh G. A. Meador K. J. Ding M. (2010). Theta oscillations mediate interaction between prefrontal cortex and medial temporal lobe in human memory. Cereb. Cortex 20, 1604–1612. doi: 10.1093/cercor/bhp223, PMID: 19861635

[ref4] Arnulfo G. Hirvonen J. Nobili L. Palva S. Palva J. M. (2015). Phase and amplitude correlations in resting-state activity in human stereotactical EEG recordings. NeuroImage 112, 114–127. doi: 10.1016/j.neuroimage.2015.02.031, PMID: 25721426

[ref5] Axmacher N. Cohen M. X. Fell J. Haupt S. Dümpelmann M. Elger C. E.. (2010). Intracranial EEG correlates of expectancy and memory formation in the human Hippocampus and nucleus Accumbens. Neuron 65, 541–549. doi: 10.1016/j.neuron.2010.02.006, PMID: 20188658

[ref6] Axmacher N. Elger C. E. Fell J. (2009). Working memory-related hippocampal deactivation interferes with long-term memory formation. J. Neurosci. 29, 1052–1060. doi: 10.1523/JNEUROSCI.5277-08.2009, PMID: 19176814 PMC6665142

[ref7] Barborica A. Mindruta I. López-Madrona V. J. Alario F. X. Trébuchon A. Donos C.. (2023). Studying memory processes at different levels with simultaneous depth and surface EEG recordings. Front. Hum. Neurosci. 17:1154038. doi: 10.3389/fnhum.2023.1154038, PMID: 37082152 PMC10110965

[ref9002] Bartlett F. C. (1933). Remembering: a study in experimental and social psychology. Br. J. Educ. Psychol. 3, 187–192. doi: 10.1111/j.2044-8279.1933.tb02913.x

[ref8] Benke T. Köylü B. Visani P. Karner E. Brenneis C. Bartha L.. (2006). Language lateralization in temporal lobe epilepsy: a comparison between fMRI and the Wada test. Epilepsia 47, 1308–1319. doi: 10.1111/j.1528-1167.2006.00549.x, PMID: 16922875

[ref9] Bernhardt B. C. Fadaie F. Liu M. Caldairou B. Gu S. Jefferies E.. (2019). Temporal lobe epilepsy: hippocampal pathology modulates connectome topology and controllability. Neurology 92, E2209–E2220. doi: 10.1212/WNL.0000000000007447, PMID: 31004070 PMC6537128

[ref10] Bragin A. Jandó G. Nádasdy Z. Hetke J. Wise K. Buzsáki G. (1995). Gamma (40-100 Hz) oscillation in the hippocampus of the behaving rat. J. Neurosci. 15, 47–60. doi: 10.1523/jneurosci.15-01-00047.1995, PMID: 7823151 PMC6578273

[ref11] Brodt S. Pöhlchen D. Flanagin V. L. Glasauer S. Gais S. Schönauer M. (2016). Rapid and independent memory formation in the parietal cortex. Proc. Natl. Acad. Sci. USA 113, 13251–13256. doi: 10.1073/pnas.1605719113, PMID: 27803331 PMC5135314

[ref12] Buck S. Sidhu M. K. (2020). A guide to designing a memory fMRI paradigm for pre-surgical evaluation in temporal lobe epilepsy. Front. Neurol. 10:1354. doi: 10.3389/fneur.2019.01354, PMID: 31998216 PMC6962296

[ref13] Buckner R. L. Koutstaal W. (1998). Functional neuroimaging studies of encoding, priming, and explicit memory retrieval. Proc. Natl. Acad. Sci. USA 95, 891–898. doi: 10.1073/pnas.95.3.891, PMID: 9448256 PMC33813

[ref14] Burke J. F. Long N. M. Zaghloul K. A. Sharan A. D. Sperling M. R. Kahana M. J. (2014). Human intracranial high-frequency activity maps episodic memory formation in space and time. NeuroImage 85, 834–843. doi: 10.1016/j.neuroimage.2013.06.067, PMID: 23827329 PMC4289670

[ref15] Busch N. A. Herrmann C. S. Müller M. M. Lenz D. Gruber T. (2006). A cross-laboratory study of event-related gamma activity in a standard object recognition paradigm. NeuroImage 33, 1169–1177. doi: 10.1016/j.neuroimage.2006.07.034, PMID: 17023180

[ref16] Buzsáki G. Anastassiou C. A. Koch C. (2012). The origin of extracellular fields and currents — EEG, ECoG, LFP and spikes. Nat. Rev. Neurosci. 13, 407–420. doi: 10.1038/nrn3241, PMID: 22595786 PMC4907333

[ref17] Castelhano J. Duarte I. Bernardino I. Pelle F. Francione S. Sales F.. (2022). Intracranial recordings in humans reveal specific hippocampal spectral and dorsal vs. ventral connectivity signatures during visual, attention and memory tasks. Sci. Rep. 12, 3488–3410. doi: 10.1038/s41598-022-07225-0, PMID: 35241722 PMC8894428

[ref18] Cohen M. X. (2019). Analyzing neural time series data. London: The MIT Press.

[ref19] Colgin L. L. (2015). Do slow and fast gamma rhythms correspond to distinct functional states in the hippocampal network? Brain Res. 1621, 309–315. doi: 10.1016/j.brainres.2015.01.005, PMID: 25591484 PMC4499490

[ref20] Colgin L. L. Denninger T. Fyhn M. Hafting T. Bonnevie T. Jensen O.. (2009). Frequency of gamma oscillations routes flow of information in the hippocampus. Nature 462, 353–357. doi: 10.1038/nature08573, PMID: 19924214

[ref21] Dalton M. A. D’souza A. Lv J. Calamante F. (2022). New insights into anatomical connectivity along the anterior–posterior axis of the human hippocampus using in vivo quantitative fibre tracking. eLife 11, 1–29. doi: 10.7554/ELIFE.76143, PMID: 36345716 PMC9643002

[ref22] Davis C. P. Altmann G. T. M. Yee E. (2020). Situational systematicity: A role for schema in understanding the differences between abstract and concrete concepts. Cogn. Neuropsychol. 37, 142–153. doi: 10.1080/02643294.2019.1710124, PMID: 31900045

[ref23] Davis C. P. Yee E. (2019). Features, labels, space, and time: factors supporting taxonomic relationships in the anterior temporal lobe and thematic relationships in the angular gyrus. Lang. Cognit. Neurosci. 34, 1347–1357. doi: 10.1080/23273798.2018.1479530

[ref24] de Andrade Morange D. Laguitton V. Carron R. Schön D. Bénar C. G. Giusiano B.. (2022). Hippocampal intracerebral evoked potentials as a marker of its functionality in drug-resistant epilepsy. Neurophysiol. Clin. 52, 323–332. doi: 10.1016/j.neucli.2022.07.001, PMID: 35989149

[ref25] de Kloet E. R. Meijer O. C. de Nicola A. F. de Rijk R. H. Joëls M. (2018). Importance of the brain corticosteroid receptor balance in metaplasticity, cognitive performance and neuro-inflammation. Front. Neuroendocrinol. 49, 124–145. doi: 10.1016/j.yfrne.2018.02.003, PMID: 29428549

[ref26] Dorey R. Piérard C. Shinkaruk S. Tronche C. Chauveau F. Baudonnat M.. (2011). Membrane mineralocorticoid but not glucocorticoid receptors of the dorsal hippocampus mediate the rapid effects of corticosterone on memory retrieval. Neuropsychopharmacology 36, 2639–2649. doi: 10.1038/npp.2011.152, PMID: 21814189 PMC3230488

[ref27] Duncan J. S. Winston G. P. Koepp M. J. Ourselin S. (2016). Brain imaging in the assessment for epilepsy surgery. Lancet Neurol. 15, 420–433. doi: 10.1016/S1474-4422(15)00383-X, PMID: 26925532 PMC6736670

[ref28] Engel A. K. Konig P. Gray C. M. Singer W. (1990). Stimulus-dependent neuronal oscillations in cat visual cortex: inter-columnar interaction as determined by cross-correlation analysis. Eur. J. Neurosci. 2, 588–606. doi: 10.1111/j.1460-9568.1990.tb00449.x, PMID: 12106294

[ref29] Fan L. Li H. Zhuo J. Zhang Y. Wang J. Chen L.. (2016). The human Brainnetome Atlas: A new brain Atlas based on connectional architecture. Cereb. Cortex 26, 3508–3526. doi: 10.1093/cercor/bhw157, PMID: 27230218 PMC4961028

[ref30] Fedele T. Tzovara A. Steiger B. Hilfiker P. Grunwald T. Stieglitz L.. (2020). The relation between neuronal firing, local field potentials and hemodynamic activity in the human amygdala in response to aversive dynamic visual stimuli. NeuroImage 213:116705. doi: 10.1016/j.neuroimage.2020.116705, PMID: 32165266

[ref31] Fell J. Klaver P. Elfadil H. Schaller C. Elger C. E. Fernández G. (2003). Rhinal-hippocampal theta coherence during declarative memory formation: interaction with gamma synchronization? Eur. J. Neurosci. 17, 1082–1088. doi: 10.1046/j.1460-9568.2003.02522.x, PMID: 12653984

[ref32] Fell J. Klaver P. Lehnertz K. Grunwald T. Schaller C. Elger C. E.. (2001). Human memory formation is accompanied by rhinal–hippocampal coupling and decoupling. Nat. Neurosci. 4, 1259–1264. doi: 10.1038/nn759, PMID: 11694886

[ref9003] Fernández G. Morris R. G. M. (2018). Memory, novelty and prior knowledge. Trends Neurosci. 41, 654–659. doi: 10.1016/j.tins.2018.08.006 30274601

[ref34] Foster B. L. Dastjerdi M. Parvizi J. (2012). Neural populations in human posteromedial cortex display opposing responses during memory and numerical processing. Proc. Natl. Acad. Sci. USA 109, 15514–15519. doi: 10.1073/pnas.1206580109, PMID: 22949666 PMC3458396

[ref35] Foster B. L. Kaveh A. Dastjerdi M. Miller K. J. Parvizi J. (2013). Human retrosplenial cortex displays transient theta phase locking with medial temporal cortex prior to activation during autobiographical memory retrieval. J. Neurosci. 33, 10439–10446. doi: 10.1523/JNEUROSCI.0513-13.2013, PMID: 23785155 PMC3685837

[ref9004] Frank D. Montaldi D. Wittmann B. Talmi D. (2018). Beneficial and detrimental effects of schema incongruence on memory for contextual events. Learn. Mem. 25, 352–360. doi: 10.1101/lm.047738.118 30012880 PMC6049394

[ref36] Fritch H. A. MacEvoy S. P. Thakral P. P. Jeye B. M. Ross R. S. Slotnick S. D. (2020). The anterior hippocampus is associated with spatial memory encoding. Brain Res. 1732:146696. doi: 10.1016/j.brainres.2020.146696, PMID: 32014532

[ref37] Fritch H. A. Spets D. S. Slotnick S. D. (2021). Functional connectivity with the anterior and posterior hippocampus during spatial memory. Hippocampus 31, 658–668. doi: 10.1002/hipo.23283, PMID: 33207019

[ref9005] Ghosh V. E. Gilboa A. (2014). What is a memory schema? A historical perspective on current neuroscience literature. Neuropsychologia. 53, 104–114. doi: 10.1016/j.neuropsychologia.2013.11.010 24280650

[ref9006] Gilboa A. Marlatte H. (2017). Neurobiology of schemas and schema-mediated memory. Trends Cogn. Sci. 21, 618–631. doi: 10.1016/j.tics.2017.04.013 28551107

[ref38] Golby A. J. Poldrack R. A. Illes J. Chen D. Desmond J. E. Gabrieli J. D. E. (2002). Memory lateralization in medial temporal lobe epilepsy assessed by functional MRI. Epilepsia 43, 855–863. doi: 10.1046/j.1528-1157.2002.20501.x, PMID: 12181004

[ref39] Grady C. L. (2020). Meta-analytic and functional connectivity evidence from functional magnetic resonance imaging for an anterior to posterior gradient of function along the hippocampal axis. Hippocampus 30, 456–471. doi: 10.1002/hipo.23164, PMID: 31589003

[ref40] Griffiths B. J. Parish G. Roux F. Michelmann S. van der Plas M. Kolibius L. D.. (2019). Directional coupling of slow and fast hippocampal gamma with neocortical alpha/beta oscillations in human episodic memory. Proc. Natl. Acad. Sci. USA 116, 21834–21842. doi: 10.1073/pnas.1914180116, PMID: 31597741 PMC6815125

[ref42] Hanslmayr S. Staresina B. P. Bowman H. (2016). Oscillations and episodic memory: addressing the synchronization/desynchronization conundrum. Trends Neurosci. 39, 16–25. doi: 10.1016/j.tins.2015.11.004, PMID: 26763659 PMC4819444

[ref43] Hartley T. Maguire E. A. Spiers H. J. Burgess N. (2003). The well-worn route and the path less traveled: distinct neural bases of route following and wayfinding in humans. Neuron 37, 877–888. doi: 10.1016/S0896-6273(03)00095-3, PMID: 12628177

[ref44] Headley D. B. Weinberger N. M. (2011). Gamma-band activation predicts both associative memory and cortical plasticity. J. Neurosci. 31, 12748–12758. doi: 10.1523/JNEUROSCI.2528-11.2011, PMID: 21900554 PMC3180928

[ref45] Henin S. Shankar A. Hasulak N. Friedman D. Dugan P. Melloni L.. (2019). Hippocampal gamma predicts associative memory performance as measured by acute and chronic intracranial EEG. Sci. Rep. 9, 593–510. doi: 10.1038/s41598-018-37561-z, PMID: 30679734 PMC6345863

[ref46] Hoffman P. Binney R. J. Lambon Ralph M. A. (2015). Differing contributions of inferior prefrontal and anterior temporal cortex to concrete and abstract conceptual knowledge. Cortex 63, 250–266. doi: 10.1016/j.cortex.2014.09.001, PMID: 25303272 PMC4317194

[ref47] Iemi L. Gwilliams L. Samaha J. Auksztulewicz R. Cycowicz Y. M. King J. R.. (2022). Ongoing neural oscillations influence behavior and sensory representations by suppressing neuronal excitability. NeuroImage 247:118746. doi: 10.1016/j.neuroimage.2021.118746, PMID: 34875382

[ref48] Jacobs J. Kahana M. J. (2009). Neural representations of individual stimuli in humans revealed by gamma-band electrocorticographic activity. J. Neurosci. 29, 10203–10214. doi: 10.1523/JNEUROSCI.2187-09.2009, PMID: 19692595 PMC2752642

[ref49] Jiang X. Gonzalez-Martinez J. Halgren E. (2019). Posterior hippocampal spindle ripples co-occur with neocortical theta bursts and downstates-upstates, and phase-lock with parietal spindles during nrem sleep in humans. J. Neurosci. 39, 8949–8968. doi: 10.1523/JNEUROSCI.2858-18.2019, PMID: 31530646 PMC6832672

[ref50] Jobst B. C. Cascino G. D. (2015). Resective epilepsy surgery for drug-resistant focal epilepsy: a review. JAMA 313, 285–293. doi: 10.1001/jama.2014.17426, PMID: 25602999

[ref51] Johnson E. L. Knight R. T. (2015). Intracranial recordings and human memory. Curr. Opin. Neurobiol. 31, 18–25. doi: 10.1016/j.conb.2014.07.021, PMID: 25113154 PMC4326634

[ref52] Kahana M. J. (2012). Foundations of human memory. New York, NY: OUP USA.

[ref53] Kim H. (2015). Encoding and retrieval along the long axis of the hippocampus and their relationships with dorsal attention and default mode networks: the HERNET model. Hippocampus 25, 500–510. doi: 10.1002/hipo.22387, PMID: 25367784

[ref54] Kjelstrup K. B. Solstad T. Brun V. H. Hafting T. Leutgeb S. Witter M. P.. (2008). Finite scale of spatial representation in the hippocampus. Science 321, 140–143. doi: 10.1126/science.1157086, PMID: 18599792

[ref55] Klimesch W. (2012). Alpha-band oscillations, attention, and controlled access to stored information. Trends Cogn. Sci. 16, 606–617. doi: 10.1016/j.tics.2012.10.007, PMID: 23141428 PMC3507158

[ref56] Klimesch W. Sauseng P. Hanslmayr S. (2007). EEG alpha oscillations: the inhibition-timing hypothesis. Brain Res. Rev. 53, 63–88. doi: 10.1016/j.brainresrev.2006.06.003, PMID: 16887192

[ref57] Knight R. T. Eichenbaum H. (2013). Multiplexed memories: a view from human cortex. Nat. Neurosci. 16, 257–258. doi: 10.1038/nn.3341, PMID: 23434977 PMC4090684

[ref58] Leão R. N. Mikulovic S. Leão K. E. Munguba H. Gezelius H. Enjin A.. (2012). OLM interneurons differentially modulate CA3 and entorhinal inputs to hippocampal CA1 neurons. Nat. Neurosci. 15, 1524–1530. doi: 10.1038/nn.3235, PMID: 23042082 PMC3483451

[ref59] Lee H. Wang C. Deshmukh S. S. Knierim J. J. (2015). Neural population evidence of functional heterogeneity along the CA3 transverse Axis: pattern completion versus pattern separation. Neuron 87, 1093–1105. doi: 10.1016/j.neuron.2015.07.012, PMID: 26298276 PMC4548827

[ref60] Lega B. C. Burke J. F. Jacobs J. Kahana M. J. (2016). Slow-theta-to-gamma phase–amplitude coupling in human hippocampus supports the formation of new episodic memories. Cereb. Cortex 26, 268–278. doi: 10.1093/cercor/bhu232, PMID: 25316340 PMC4677977

[ref61] Lega B. C. Jacobs J. Kahana M. J. (2012). Human hippocampal theta oscillations and the formation of episodic memories. Hippocampus 22, 748–761. doi: 10.1002/hipo.20937, PMID: 21538660

[ref62] Lepage M. Habib R. Tulving E. (1998). Hippocampal PET activations of memory encoding and retrieval: the HIPER model. Hippocampus 8, 313–322. doi: 10.1002/(SICI)1098-1063(1998)8:4<313::AID-HIPO1>3.0.CO;2-I, PMID: 9744418

[ref63] Li J. Cao D. Dimakopoulos V. Shi W. Yu S. Fan L.. (2021). Anterior-posterior hippocampal dynamics support working memory processing. J. Neurosci. 22:1287. doi: 10.1523/jneurosci.1287-21.2021 PMC8802917 34819340

[ref64] Lin J. J. Umbach G. Rugg M. D. Lega B. C. (2019). Gamma oscillations during episodic memory processing provide evidence for functional specialization in the longitudinal axis of the human hippocampus. Hippocampus 29, 68–72. doi: 10.1002/hipo.23016, PMID: 30394594 PMC6519081

[ref65] Long N. M. Burke J. F. Kahana M. J. (2014). Subsequent memory effect in intracranial and scalp EEG. NeuroImage 84, 488–494. doi: 10.1016/j.neuroimage.2013.08.052, PMID: 24012858 PMC3849113

[ref66] Maris E. Oostenveld R. (2007). Nonparametric statistical testing of EEG- and MEG-data. J. Neurosci. Methods 164, 177–190. doi: 10.1016/j.jneumeth.2007.03.024, PMID: 17517438

[ref67] Marks V. S. Saboo K. V. Topçu Ç. Lech M. Thayib T. P. Nejedly P.. (2021). Independent dynamics of low, intermediate, and high frequency spectral intracranial EEG activities during human memory formation. NeuroImage 245:118637. doi: 10.1016/j.neuroimage.2021.118637, PMID: 34644594

[ref68] Martinovic J. Gruber T. Müller M. M. (2007). Induced gamma band responses predict recognition delays during object identification. J. Cogn. Neurosci. 19, 921–934. doi: 10.1162/jocn.2007.19.6.921, PMID: 17536963

[ref69] Matsumoto J. Y. Stead M. Kucewicz M. T. Matsumoto A. J. Peters P. A. Brinkmann B. H.. (2013). Network oscillations modulate interictal epileptiform spike rate during human memory. Brain 136, 2444–2456. doi: 10.1093/brain/awt159, PMID: 23803305 PMC3722348

[ref71] Mercier M. R. Dubarry A. S. Tadel F. Avanzini P. Axmacher N. Cellier D.. (2022). Advances in human intracranial electroencephalography research, guidelines and good practices. NeuroImage 260:119438. doi: 10.1016/j.neuroimage.2022.119438, PMID: 35792291 PMC10190110

[ref72] Milivojevic B. Vicente-Grabovetsky A. Doeller C. F. (2015). Insight reconfigures hippocampal-prefrontal memories. Curr. Biol. 25, 821–830. doi: 10.1016/j.cub.2015.01.033, PMID: 25728693

[ref73] Montaz-Rosset M. S. Scholly J. Voulleminot P. Severac F. Hirsch E. Valenti-Hirsch M. P.. (2019). Comparison of functional deficit zone defined by FDG PET to the epileptogenic zones described in stereo-electroencephalograph in drug-resistant epileptic patients treated by surgery. Clin. Nucl. Med. 44, 526–531. doi: 10.1097/RLU.0000000000002615, PMID: 31135520

[ref74] Mormann F. Fell J. Axmacher N. Weber B. Lehnertz K. Elger C. E.. (2005). Phase/amplitude reset and theta-gamma interaction in the human medial temporal lobe during a continuous word recognition memory task. Hippocampus 15, 890–900. doi: 10.1002/hipo.20117, PMID: 16114010

[ref75] Mormann F. Fernández G. Klaver P. Weber B. Elger C. E. Fell J. (2007). Declarative memory formation in hippocampal sclerosis: an intracranial event-related potentials study. Neuroreport 18, 317–321. doi: 10.1097/WNR.0b013e3280287ae9, PMID: 17435595

[ref76] Morris R. G. M. (2006). “Theories of hippocampal function” in The Hippocampus book. eds. Andersen P. Morris R. Amaral D. G. Bliss T. O’Keefe J. (Oxford: Oxford University Press), 581–713.

[ref77] Murray E. A. Wise S. P. Graham K. S. (2018). Representational specializations of the hippocampus in phylogenetic perspective. Neurosci. Lett. 680, 4–12. doi: 10.1016/j.neulet.2017.04.065, PMID: 28473258 PMC5665731

[ref9007] Nadel L. Campbell J. Ryan L. R. (2007). Autobiographical memory retrieval and hippocampal activation as a function of repetition and the passage of time. Neural Plast. 2007, 1–14. doi: 10.1155/2007/90472 PMC2233815 18274617

[ref78] Nadel L. Hoscheidt S. Ryan L. R. (2013). Spatial cognition and the hippocampus: the anterior-posterior axis. J. Cogn. Neurosci. 25, 22–28. doi: 10.1162/jocn_a_00313, PMID: 23198887

[ref79] Nahum L. Gabriel D. Spinelli L. Momjian S. Seeck M. Michel C. M.. (2011). Rapid consolidation and the human hippocampus: intracranial recordings confirm surface EEG. Hippocampus 21, 689–693. doi: 10.1002/hipo.20819, PMID: 20865742

[ref80] Norman Y. Yeagle E. M. Khuvis S. Harel M. Mehta A. D. Malach R. (2019). Hippocampal sharp-wave ripples linked to visual episodic recollection in humans. Science 365:aax1030. doi: 10.1126/science.aax1030, PMID: 31416934

[ref81] Oostenveld R. Fries P. Maris E. Schoffelen J.-M. (2011). FieldTrip: open source software for advanced analysis of MEG, EEG, and invasive electrophysiological data. Comput. Intell. Neurosci. 2011, 1–9. doi: 10.1155/2011/156869, PMID: 21253357 PMC3021840

[ref9008] Packard P. A. Steiger T. K. Fuentemilla L. Bunzeck N. (2020). Neural oscillations and event-related potentials reveal how semantic congruence drives long-term memory in both young and older humans. Sci. Rep. 10, 1–17. doi: 10.1038/s41598-020-65872-7 32499519 PMC7272459

[ref82] Parvizi J. Kastner S. (2018). Promises and limitations of human intracranial electroencephalography. Nat. Neurosci. 21, 474–483. doi: 10.1038/s41593-018-0108-2, PMID: 29507407 PMC6476542

[ref9009] Piaget J. (2003). The psychology of intelligence. Routledge.

[ref83] Poppenk J. Evensmoen H. R. Moscovitch M. Nadel L. (2013). Long-axis specialization of the human hippocampus. Trends Cogn. Sci. 17, 230–240. doi: 10.1016/j.tics.2013.03.005, PMID: 23597720

[ref84] Poppenk J. Köhler S. Moscovitch M. (2010). Revisiting the novelty effect: when familiarity, not novelty, enhances memory. J. Exp. Psychol. Learn. Mem. Cogn. 36, 1321–1330. doi: 10.1037/a0019900, PMID: 20804299

[ref85] Ranganath C. Yonelinas A. P. Cohen M. X. Dy C. J. Tom S. M. D’Esposito M. (2004). Dissociable correlates of recollection and familiarity within the medial temporal lobes. Neuropsychologia 42, 2–13. doi: 10.1016/j.neuropsychologia.2003.07.006, PMID: 14615072

[ref86] Rutishauser U. Schuman E. M. Mamelak A. N. (2008). Activity of human hippocampal and amygdala neurons during retrieval of declarative memories. Proc. Natl. Acad. Sci. USA 105, 329–334. doi: 10.1073/pnas.0706015105, PMID: 18162554 PMC2224211

[ref87] Schacher M. Haemmerle B. Woermann F. G. Okujava M. Huber D. Grunwald T.. (2006). Amygdala fMRI lateralizes temporal lobe epilepsy. Neurology 66, 81–87. doi: 10.1212/01.wnl.0000191303.91188.00, PMID: 16401851

[ref88] Sederberg P. B. Kahana M. J. Howard M. W. Donner E. J. Madsen J. R. (2003). Theta and gamma oscillations during encoding predict subsequent recall. J. Neurosci. 23, 10809–10814. doi: 10.1523/JNEUROSCI.23-34-10809.2003, PMID: 14645473 PMC6740970

[ref89] Sederberg P. B. Schulze-Bonhage A. Madsen J. R. Bromfield E. B. Litt B. Brandt A.. (2007). Gamma oscillations distinguish true from false memories. Psychol. Sci. 18, 927–932. doi: 10.1111/j.1467-9280.2007.02003.x, PMID: 17958703 PMC2897900

[ref90] Sederberg P. B. Schulze-Bonhage A. Madsen J. R. Bromfield E. B. McCarthy D. C. Brandt A.. (2006). Hippocampal and neocortical gamma oscillations predict memory formation in humans. Cereb. Cortex 17, 1190–1196. doi: 10.1093/cercor/bhl030, PMID: 16831858

[ref91] Shamshiri E. A. Sheybani L. Vulliemoz S. (2019). The role of EEG-fMRI in studying cognitive network alterations in epilepsy. Front. Neurol. 10:1033. doi: 10.3389/fneur.2019.01033, PMID: 31608007 PMC6771300

[ref92] Snodgrass J. G. Corwin J. (1988). Pragmatics of measuring recognition memory: applications to dementia and amnesia. J. Exp. Psychol. Gen. 117, 34–50. doi: 10.1037/0096-3445.117.1.34, PMID: 2966230

[ref93] Staresina B. P. Cooper E. Henson R. N. (2013). Reversible information flow across the medial temporal lobe: the hippocampus links cortical modules during memory retrieval. J. Neurosci. 33, 14184–14192. doi: 10.1523/JNEUROSCI.1987-13.2013, PMID: 23986252 PMC3756762

[ref94] Staresina B. P. Davachi L. (2006). Differential encoding mechanisms for subsequent associative recognition and free recall. J. Neurosci. 26, 9162–9172. doi: 10.1523/JNEUROSCI.2877-06.2006, PMID: 16957073 PMC6674493

[ref95] Staresina B. P. Fell J. Lam A. T. A. Do A. Henson R. N. Do Lam A. T. A.. (2012). Memory signals are temporally dissociated in and across human hippocampus and perirhinal cortex. Nat. Neurosci. 15, 1167–1173. doi: 10.1038/nn.3154, PMID: 22751037 PMC3428860

[ref96] Staresina B. P. Michelmann S. Bonnefond M. Jensen O. Axmacher N. Fell J. (2016). Hippocampal pattern completion is linked to gamma power increases and alpha power decreases during recollection. eLife 5, 1–18. doi: 10.7554/elife.17397, PMID: 27508355 PMC4980114

[ref97] Steinvorth S. Wang C. Ulbert I. Schomer D. Halgren E. (2010). Human entorhinal gamma and theta oscillations selective for remote autobiographical memory. Hippocampus 20, 166–173. doi: 10.1002/hipo.20597, PMID: 19338019

[ref98] Stolk A. Griffin S. van der Meij R. Dewar C. Saez I. Lin J. J.. (2018). Integrated analysis of anatomical and electrophysiological human intracranial data. Nat. Protoc. 13, 1699–1723. doi: 10.1038/s41596-018-0009-6, PMID: 29988107 PMC6548463

[ref99] Strange B. A. Witter M. P. Lein E. S. Moser E. I. (2014). Functional organization of the hippocampal longitudinal axis. Nat. Rev. Neurosci. 15, 655–669. doi: 10.1038/nrn3785, PMID: 25234264

[ref100] Sullivan M. A. Fritch H. A. Slotnick S. D. (2024). Spatial memory encoding is associated with the anterior and posterior hippocampus: an fMRI activation likelihood estimation meta-analysis. Hippocampus 34, 1–8. doi: 10.1002/hipo.23632 39150234

[ref101] Supp G. G. Schlö Gl A. Trujillo-Barreto N. Mü Ller M. M. Gruber T. Schlögl A.. (2007). Directed cortical information flow during human object recognition: analyzing induced EEG gamma-band responses in Brain’s source space. PLoS One 2:e684. doi: 10.1371/journal.pone.0000684, PMID: 17668062 PMC1925146

[ref102] Sweeney-Reed C. M. Zaehle T. Voges J. Schmitt F. C. Buentjen L. Kopitzki K.. (2016). Pre-stimulus thalamic theta power predicts human memory formation. NeuroImage 138, 100–108. doi: 10.1016/j.neuroimage.2016.05.042, PMID: 27208861

[ref103] Tadel F. Baillet S. Mosher J. C. Pantazis D. Leahy R. M. (2011). Brainstorm: A user-friendly application for MEG/EEG analysis. Comput. Intell. Neurosci. 2011, 1–13. doi: 10.1155/2011/879716, PMID: 21584256 PMC3090754

[ref104] Thompson C. L. Pathak S. D. Jeromin A. Ng L. L. MacPherson C. R. Mortrud M. T.. (2008). Genomic anatomy of the Hippocampus. Neuron 60, 1010–1021. doi: 10.1016/j.neuron.2008.12.008, PMID: 19109908

[ref105] Tort A. B. L. Komorowski R. W. Manns J. R. Kopell N. J. Eichenbaum H. (2009). Theta-gamma coupling increases during the learning of item-context associations. Proc. Natl. Acad. Sci. USA 106, 20942–20947. doi: 10.1073/pnas.0911331106, PMID: 19934062 PMC2791641

[ref9010] Toyota H. (1996). Effects of Semantic and syntactic congruity on incidental free recall in japanese sentences. Percept. Mot. Skills. 82, 811–816. doi: 10.2466/pms.1996.82.3.811

[ref106] Tse D. Langston R. F. Kakeyama M. Bethus I. Spooner P. A. Wood E. R.. (2007). Schemas and memory consolidation. Science 316, 76–82. doi: 10.1126/science.1135935, PMID: 17412951

[ref107] Tsukiura T. Fujii T. Takahashi T. Xiao R. Sugiura M. Okuda J.. (2002). Medial temporal lobe activation during context-dependent relational processes in episodic retrieval: an fMRI study. Hum. Brain Mapp. 17, 203–213. doi: 10.1002/hbm.10068, PMID: 12395388 PMC6871954

[ref108] Tulving E. Markowitsch H. J. Craik F. I. M. Habib R. Houle S. (1996). Novelty and familiarity activations in PET studies of memory encoding and retrieval. Cereb. Cortex 6, 71–79. doi: 10.1093/cercor/6.1.71, PMID: 8670640

[ref9011] van der Linden M. Berkers R. M. W. J. Morris R. G. M. Fernández G. (2017). Angular gyrus involvement at encoding and retrieval is associated with durable but less specific memories. J. Neurosci. 37, 9474–9485. doi: 10.1523/JNEUROSCI.3603-16.2017 28871031 PMC6596768

[ref109] van Kesteren M. T. R. Beul S. F. Takashima A. Henson R. N. Ruiter D. J. Fernández G. (2013). Differential roles for medial prefrontal and medial temporal cortices in schema-dependent encoding: from congruent to incongruent. Neuropsychologia 51, 2352–2359. doi: 10.1016/j.neuropsychologia.2013.05.027, PMID: 23770537

[ref110] van Kesteren M. T. R. Rijpkema M. Ruiter D. J. Fernández G. Fernandez G. (2010). Retrieval of associative information congruent with prior knowledge is related to increased medial prefrontal activity and connectivity. J. Neurosci. 30, 15888–15894. doi: 10.1523/JNEUROSCI.2674-10.2010, PMID: 21106827 PMC6633736

[ref111] van Kesteren M. T. R. Ruiter D. J. Fernández G. Henson R. N. (2012). How schema and novelty augment memory formation. Trends Neurosci. 35, 211–219. doi: 10.1016/j.tins.2012.02.001, PMID: 22398180

[ref112] Vila-Vidal M. Khawaja M. Carreño M. Roldán P. Rumià J. Donaire A.. (2023). Assessing the coupling between local neural activity and global connectivity fluctuations: application to human intracranial electroencephalography during a cognitive task. Hum. Brain Mapp. 44, 1173–1192. doi: 10.1002/hbm.26150, PMID: 36437716 PMC9875936

[ref113] Vinton A. B. Carne R. Hicks R. J. Desmond P. M. Kilpatrick C. Kaye A. H.. (2007). The extent of resection of FDG-PET hypometabolism relates to outcome of temporal lobectomy. Brain 130, 548–560. doi: 10.1093/brain/awl232, PMID: 16959818

[ref114] Von Stein A. Sarnthein J. (2000). Different frequencies for different scales of cortical integration: from local gamma to long range alpha/theta synchronization. Int. J. Psychophysiol. 38, 301–313. doi: 10.1016/S0167-8760(00)00172-0, PMID: 11102669

[ref115] Wang D. X. Ng N. Seger S. E. Ekstrom A. D. Kriegel J. L. Lega B. C. (2023). Machine learning classifiers for electrode selection in the design of closed-loop neuromodulation devices for episodic memory improvement. Cereb. Cortex 33, 8150–8163. doi: 10.1093/cercor/bhad105, PMID: 36997155 PMC10321120

[ref116] Watrous A. J. Tandon N. Conner C. R. Pieters T. A. Ekstrom A. D. (2013). Frequency-specific network connectivity increases underlie accurate spatiotemporal memory retrieval. Nat. Neurosci. 16, 349–356. doi: 10.1038/nn.3315, PMID: 23354333 PMC3581758

[ref117] Wong C. H. Bleasel A. Wen L. Eberl S. Byth K. Fulham M.. (2010). The topography and significance of extratemporal hypometabolism in refractory mesial temporal lobe epilepsy examined by FDG-PET. Epilepsia 51, 1365–1373. doi: 10.1111/j.1528-1167.2010.02552.x, PMID: 20384730

[ref118] Youngerman B. E. Khan F. A. McKhann G. M. (2019). Stereoelectroencephalography in epilepsy, cognitive neurophysiology, and psychiatric disease: safety, efficacy, and place in therapy. Neuropsychiatr. Dis. Treat. 15, 1701–1716. doi: 10.2147/NDT.S177804, PMID: 31303757 PMC6610288

