# Supplementary material for: Functional segregation of rostral and caudal hippocampus in associative memory
Source: Front Hum Neurosci. 2025 Feb 10;19:1509163. doi: 10.3389/fnhum.2025.1509163 (PMC11848949; doi:10.3389/fnhum.2025.1509163)
Supplement: Supplementary file 1 [file Data_Sheet_1.pdf]

Supplementary Table 1. Localization and anatomical labeling of contacts mapped according to the protocol outlined in Stolk et al. (2018), using the Brainnetome Atlas (Fan et al., 2016).

Encoding

Patient	Contact	x	y	z	location
1	mAHL2	-30.48709749	-12.12159308	-14.9700458	'Hipp, Left Hippocampus rHipp, rostral hippocampus'
1	mPHR1	33.72471741	-31.49064114	-10.37379102	'Hipp, Right Hippocampus cHipp, caudal hippocampus'
2	mAHL1	-30.85439073	-11.94073715	-15.69944995	'Hipp, Left Hippocampus cHipp, caudal hippocampus'
2	mAHL2	-33.91792443	-12.73074683	-15.88886052	'Hipp, Left Hippocampus cHipp, caudal hippocampus'
2	mAHR1	33.4505689	-13.87934655	-16.35714876	'Hipp, Right Hippocampus rHipp, rostral hippocampus'
2	mAHR2	36.66149035	-15.10409023	-16.02814205	'Hipp, Right Hippocampus rHipp, rostral hippocampus'
2	mECL1	-26.1918036	-7.611772368	-28.75179057	Hipp, Left Hippocampus rHipp, rostral hippocampus'
2	mECL2	-30.16644066	-7.607709982	-28.18966015	'Hipp, Left Hippocampus rHipp, rostral hippocampus'
2	mECR1	25.21400777	-8.053176647	-29.27431369	'Hipp, Right Hippocampus rHipp, rostral hippocampus'
2	mPHL1	-35.00473446	-29.20849371	-7.320541711	'Hipp, Left Hippocampus cHipp, caudal hippocampus'
3	mAHL1	-30.1874843	-20.93108758	-14.66286856	'Hipp, Left Hippocampus cHipp, caudal hippocampus'
3	mAHL2	-33.04524898	-20.98115898	-14.62515754	'Hipp, Left Hippocampus

					cHipp, caudal hippocampus'
3	mAL1	-25.43341369	-7.467188989	-22.6484715	'Hipp, Left Hippocampus rHipp, rostral hippocampus'
3	mAL2	-28.81448512	-6.980045897	-22.0493195	'Hipp, Left Hippocampus rHipp, rostral hippocampus'
3	mAL3	-34.4363204	-7.97934237	-20.77809605	'Hipp, Left Hippocampus rHipp, rostral hippocampus'
4	mPHR1	31.11650232	-28.74555711	-9.969029051	Hipp, Right Hippocampus cHipp, caudal hippocampus'
4	mPHR2	36.64510957	-31.31798963	-9.956249312	'Hipp, Right Hippocampus cHipp, caudal hippocampus'
5	mAHR1	34.27400898	-14.86178808	-19.96404297	'Hipp, Right Hippocampus cHipp, caudal hippocampus'
5	mPHR1	32.68767631	-30.95835294	-7.55636072	'Hipp, Right Hippocampus cHipp, caudal hippocampus'
5	mPHR2	38.32246907	-29.81522548	-6.471373578	'Hipp, Right Hippocampus cHipp, caudal hippocampus'
6	AHL1_0005	-28.97845832	-7.510735145	32.09910307	Hipp, Left Hippocampus cHipp, caudal hippocampus'
6	AHL2_0005	-32.09707253	-6.952404923	32.75940649	'Hipp, Left Hippocampus cHipp, caudal hippocampus'
6	PHL1_0005	-31.30503826	-13.13305337	29.86439244	Hipp, Left Hippocampus cHipp, caudal hippocampus'
6	PHL2_0005	-33.32038919	-12.49191146	31.17456747	Hipp, Left Hippocampus cHipp, caudal hippocampus'

6	PHR2_0005	16.7040658	-27.12650145	16.75268568	Hipp, Right Hippocampus cHipp, caudal hippocampus'

Retrieval

Patient	Contact	x	y	z	location
2	mAHL1	-30.85439073	-11.94073715	-15.69944995	'Hipp, Left Hippocampus cHipp, caudal hippocampus'
2	mAHR1	33.4505689	-13.87934655	-16.35714876	'Hipp, Right Hippocampus rHipp, rostral hippocampus'
2	mAHR2	36.66149035	-15.10409023	-16.02814205	'Hipp, Right Hippocampus rHipp, rostral hippocampus'
2	mECL1	-26.1918036	-7.611772368	-28.75179057	Hipp, Left Hippocampus rHipp, rostral hippocampus'
2	mECL2	-30.16644066	-7.607709982	-28.18966015	'Hipp, Left Hippocampus rHipp, rostral hippocampus'
2	mPHL1	-35.00473446	-29.20849371	-7.320541711	'Hipp, Left Hippocampus cHipp, caudal hippocampus'
3	mAHL1	-30.1874843	-20.93108758	-14.66286856	'Hipp, Left Hippocampus cHipp, caudal hippocampus'
3	mAHL2	-33.04524898	-20.98115898	-14.62515754	'Hipp, Left Hippocampus cHipp, caudal hippocampus'
3	mAL1	-25.43341369	-7.467188989	-22.6484715	'Hipp, Left Hippocampus rHipp, rostral hippocampus'
3	mAL2	-28.81448512	-6.980045897	-22.0493195	'Hipp, Left Hippocampus rHipp, rostral hippocampus'
3	mAL3	-34.4363204	-7.97934237	-20.77809605	'Hipp, Left Hippocampus rHipp, rostral hippocampus'
5	mAHR1	34.27400898	-14.86178808	-19.96404297	'Hipp, Right Hippocampus

					cHipp, caudal hippocampus'
5	mPHR1	32.68767631	-30.95835294	-7.55636072	'Hipp, Right Hippocampus cHipp, caudal hippocampus'
5	mPHR2	38.32246907	-29.81522548	-6.471373578	'Hipp, Right Hippocampus cHipp, caudal hippocampus'
6	AHL1_0006	-28.97845832	-7.510735145	32.09910307	Hipp, Left Hippocampus cHipp, caudal hippocampus'
6	AHL2_0006	-32.09707253	-6.952404923	32.75940649	'Hipp, Left Hippocampus cHipp, caudal hippocampus'
6	PHL1_0006	-31.30503826	-13.13305337	29.86439244	Hipp, Left Hippocampus cHipp, caudal hippocampus'
6	PHL2_0006	-33.32038919	-12.49191146	31.17456747	Hipp, Left Hippocampus cHipp, caudal hippocampus'

Supplementary Table 2. Spearman's rank-order correlations between individual subjective and objective congruence rates.

**Subject**	**Spearman's *r***	R^2	***p*-value**
**1**	0.57	0.33	< .001
**2**	0.59	0.35	< .001
**3**	0.46	0.21	< .001
**4**	0.58	0.34	< .001
**5**	0.36	0.13	< .001
**6**	0.49	0.24	< .001

Supplementary Figures Captions

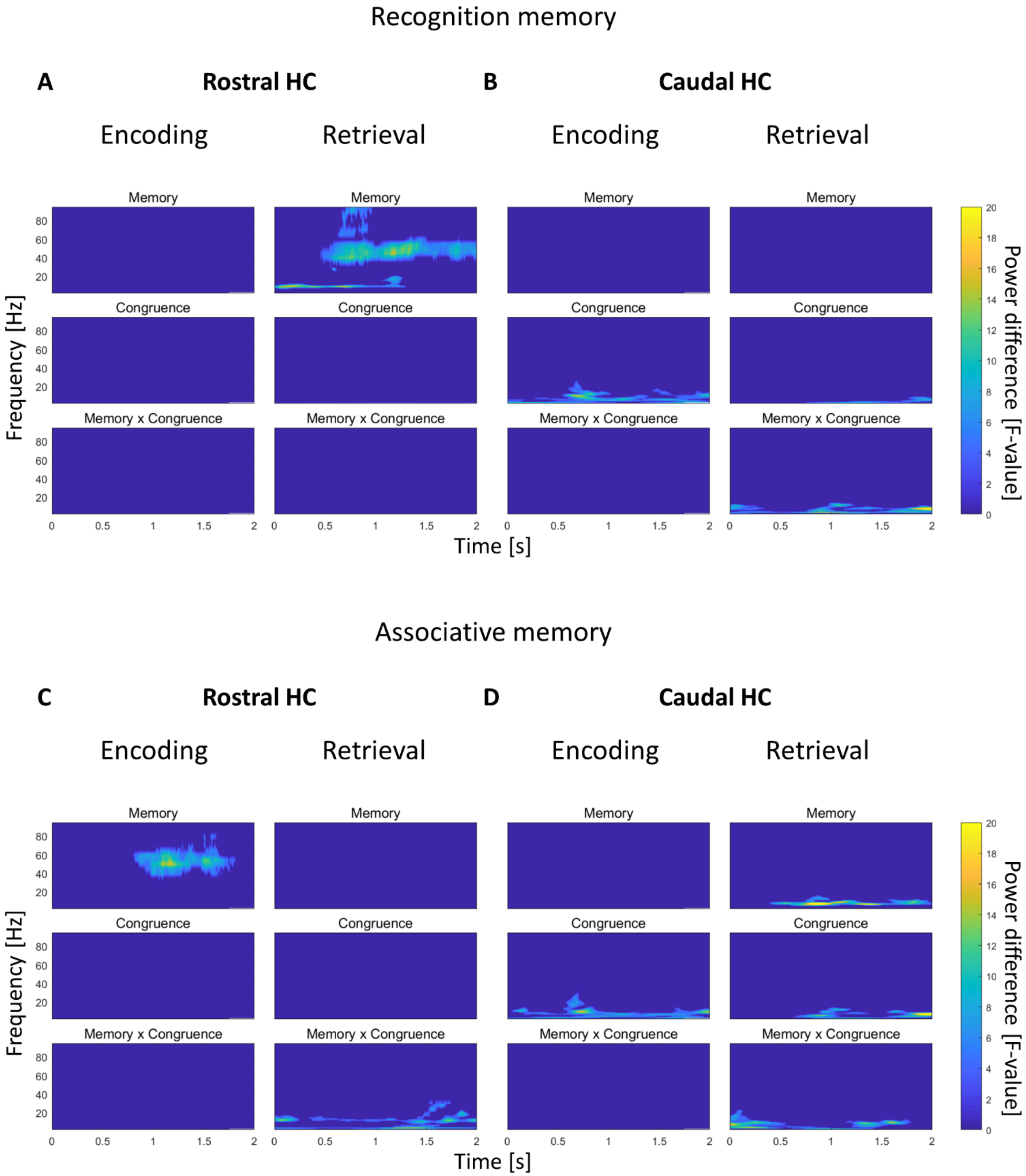

Supplementary Figure 1. *F*-maps for the GLMs relating power modulation in the time-frequency domain with behavioral factors Memory, Congruence and their mixed effect (0 corresponds to the stimulus onset). Non-significant differences are set to zero. GLM for oscillatory power with memory categorized as Hits / Misses and congruence as Congruent / Intermediate / Incongruent in **A.** rostral HC, **B.** caudal HC. GLM for oscillatory power, with memory categorized as Correctly associated (CA)

/ Incorrectly associated (IA) and congruence as Congruent / Intermediate / Incongruent in **C.** rostral HC, **D.** caudal HC. Non-significant differences are set to zero. Successful recognition at retrieval is associated with early modulation in low-frequency power and later modulation in gamma band in the rostral HC. Successful encoding of associative material is associated with late modulation in the gamma band in the rostral HC. Successful retrieval of associative material is associated with low-frequency modulation in the caudal HC. Congruence level at encoding and retrieval is associated with low-frequency modulation in the caudal HC.

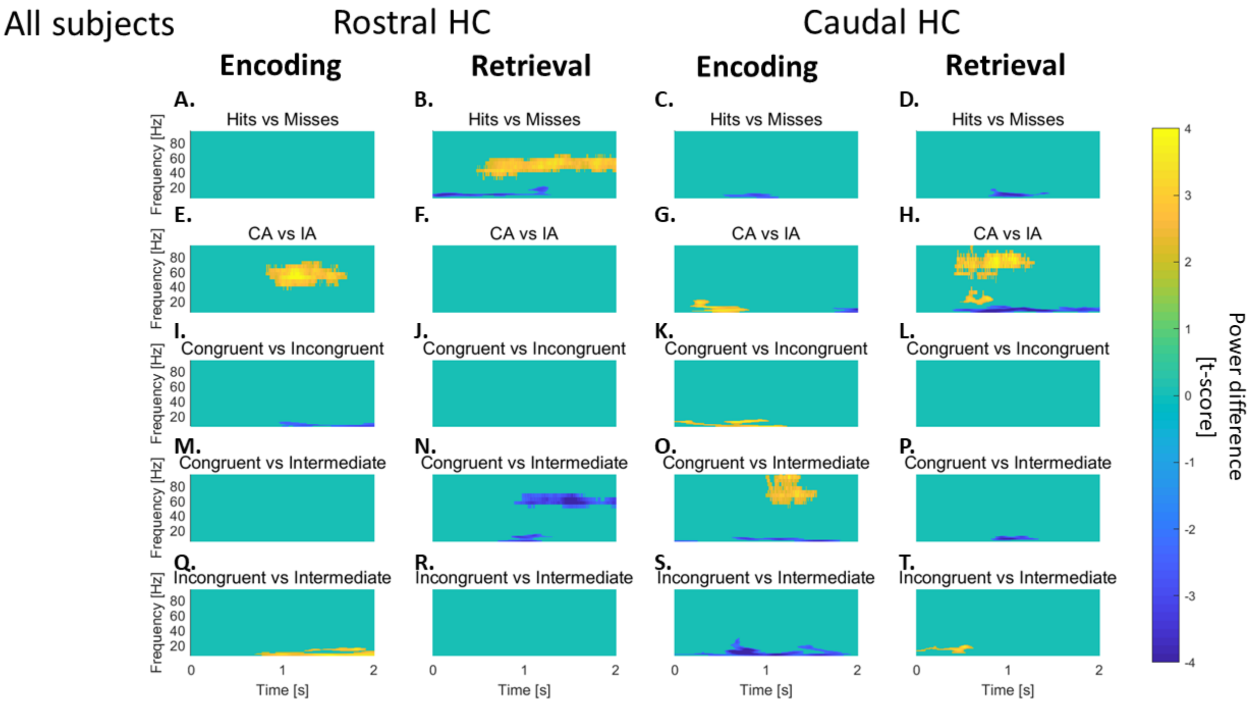

Supplementary Figure 2. Time-frequency maps showing memory-related and congruence-related power contrasts during encoding and retrieval in the rostral portion of the HC and in the caudal portion of the HC. 0 corresponds to the stimulus onset. Non-significant differences are set to zero. During encoding, the rostral HC showed a selective associative memory effect. Encoding of subsequently correctly associated items (CA) shows significantly higher gamma power increase and beta power decrease compared to the incorrectly associated items (IA). On the contrary, during retrieval rostral HC activity is selective for the recognition memory: correct item recognition was associated with higher alpha power decrease and gamma power increase compared to misses. Concurrently, during retrieval, activity patterns in the caudal HC showed selective effect for remembrance of an item and its context (CA trials) which was accompanied by higher low-frequency power decrease and higher gamma power increase compared to cases where the context was forfeit (Misses). At encoding, the caudal HC showed a selective effect for object-scene items of intermediate congruence, in the significantly lower low-frequency power decrease, compared to congruent and incongruent items.

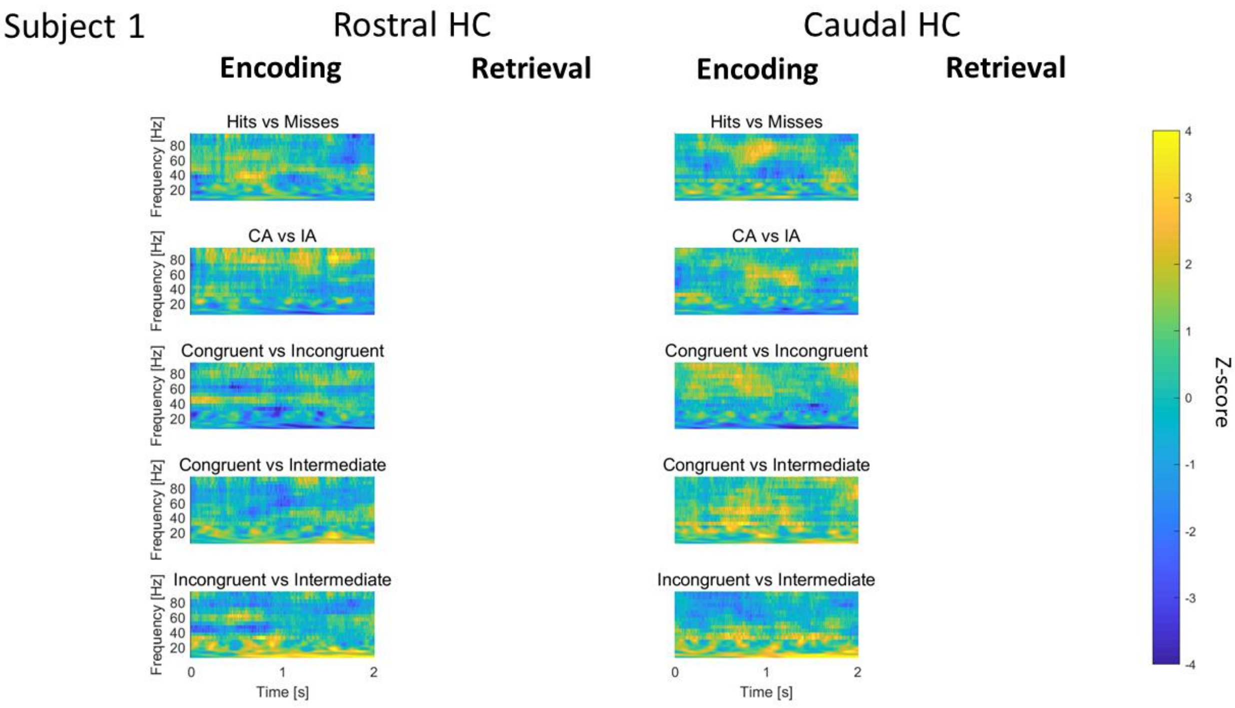
Subject 1
Rostral HC
Caudal HC
Encoding
Retrieval
Encoding
Retrieval
Hits vs Misses
CA vs IA
Congruent vs Incongruent
Congruent vs Intermediate
Incongruent vs Intermediate
Frequency [Hz]
Time [s]
Z-score

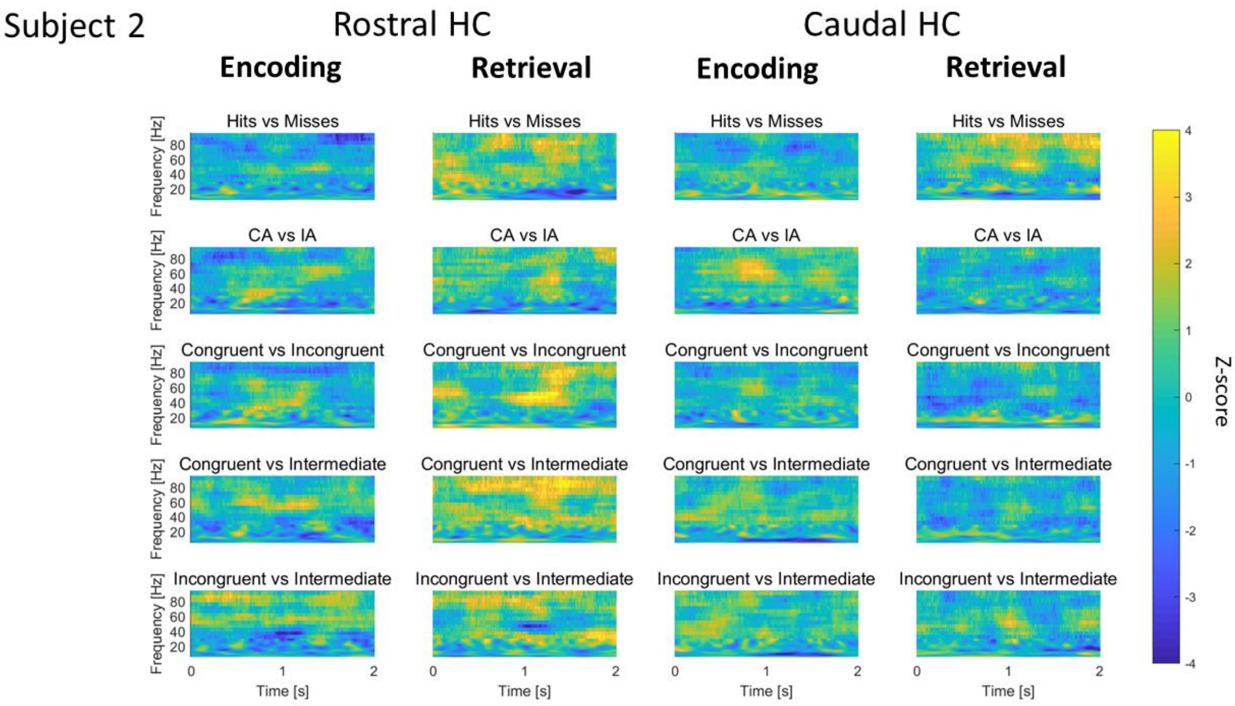
Subject 2
Rostral HC
Caudal HC
Encoding
Retrieval
Encoding
Retrieval
Hits vs Misses
CA vs IA
Congruent vs Incongruent
Congruent vs Intermediate
Incongruent vs Intermediate
Frequency [Hz]
Time [s]
Z-score

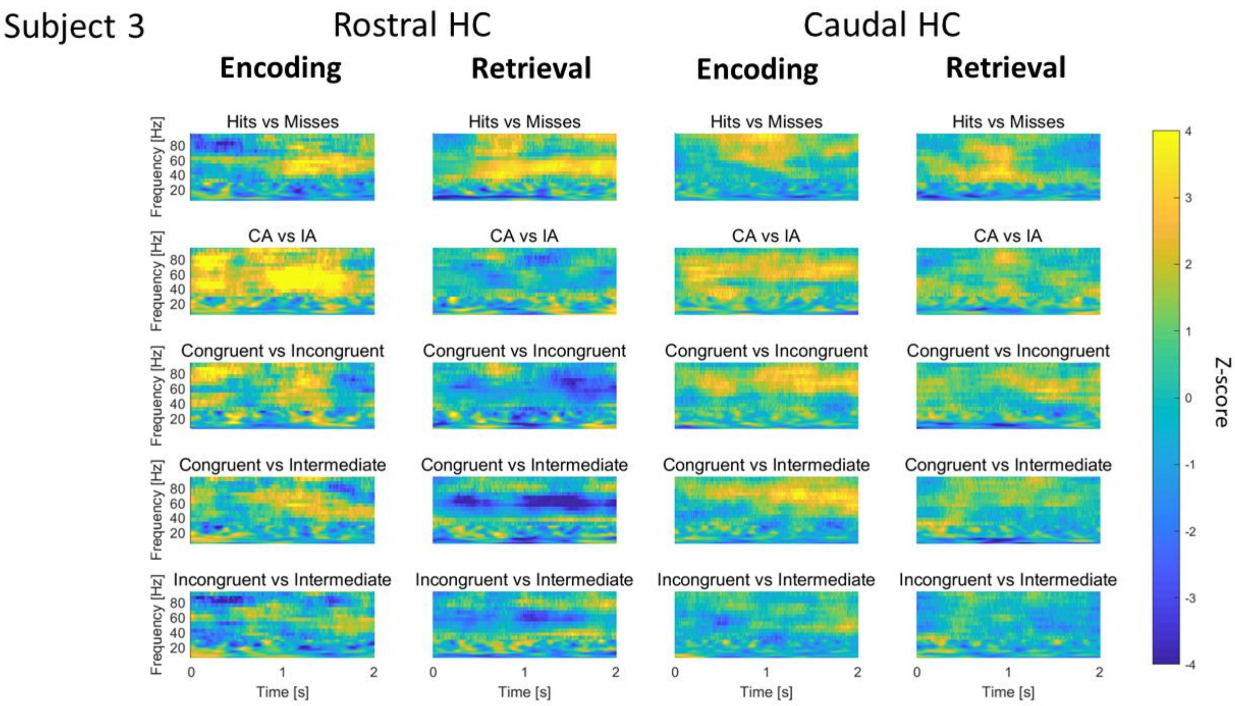
Subject 3
Rostral HC
Caudal HC
Encoding
Retrieval
Encoding
Retrieval
Hits vs Misses
CA vs IA
Congruent vs Incongruent
Congruent vs Intermediate
Incongruent vs Intermediate
Frequency [Hz]
Time [s]
Z-score

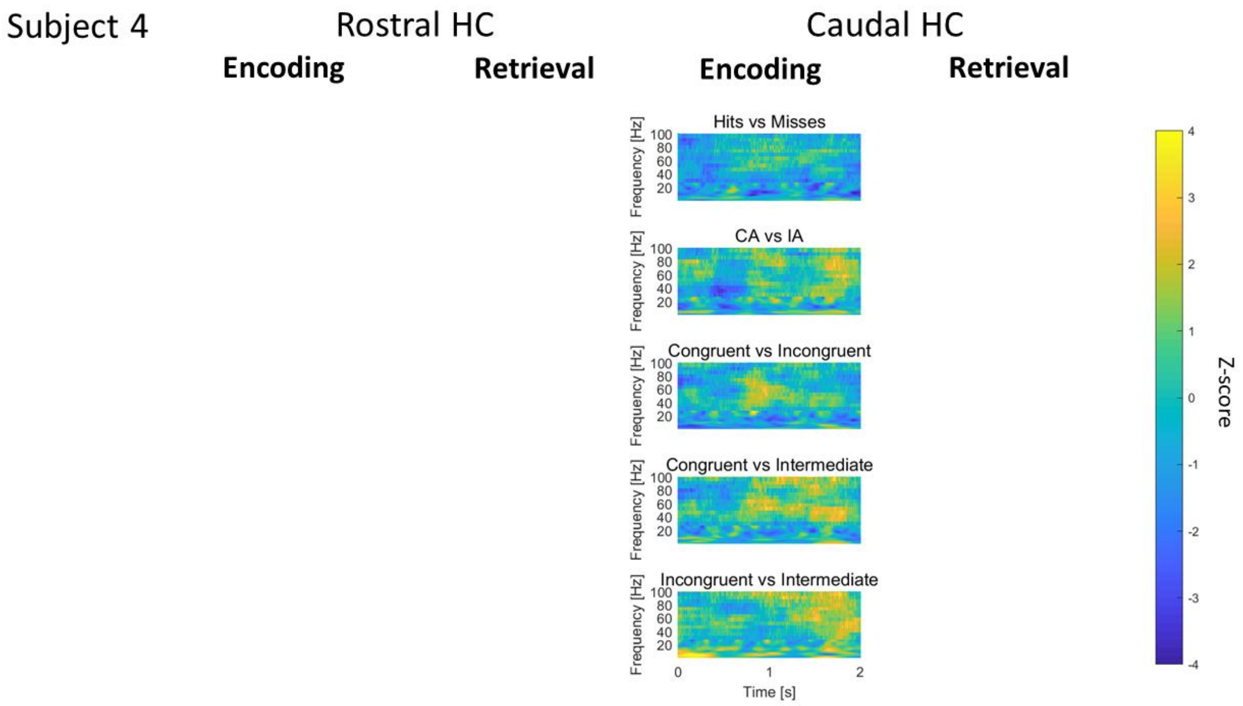
Subject 4
Rostral HC
Caudal HC
Encoding
Retrieval
Encoding
Retrieval
Hits vs Misses
CA vs IA
Congruent vs Incongruent
Congruent vs Intermediate
Incongruent vs Intermediate
Frequency [Hz]
Time [s]
Z-score

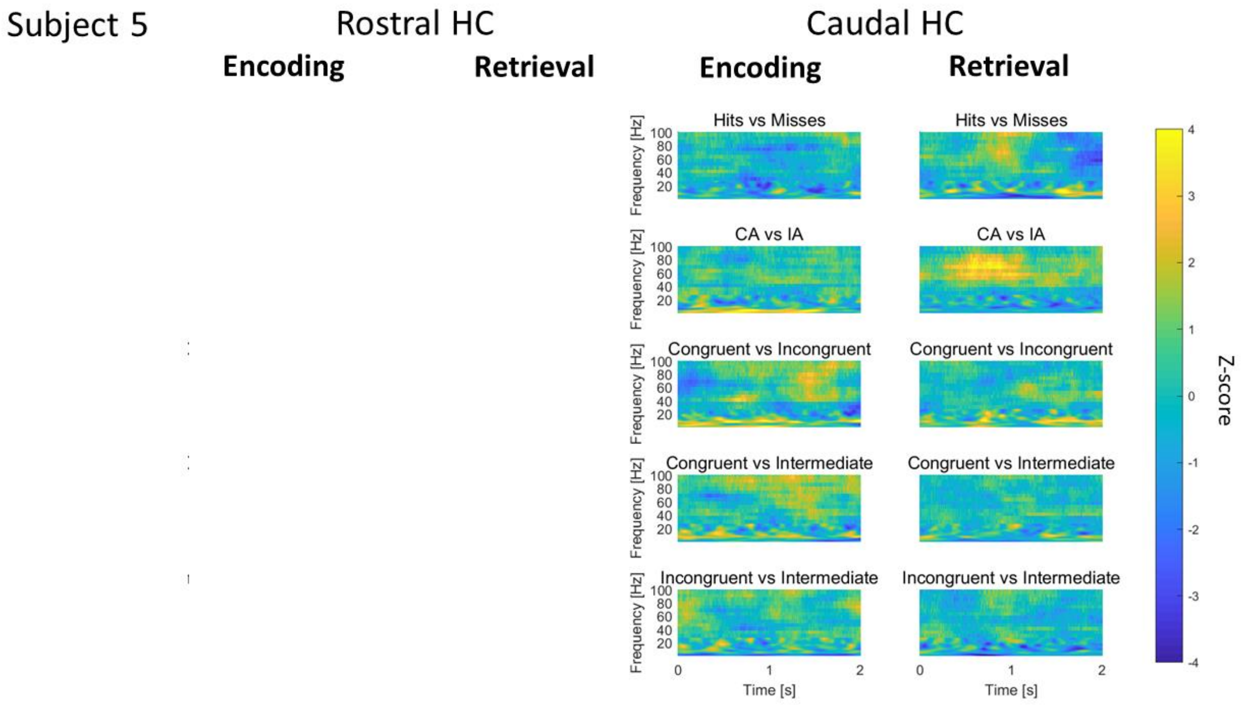
Subject 5
Rostral HC
Caudal HC
Encoding
Retrieval
Encoding
Retrieval
Hits vs Misses
CA vs IA
Congruent vs Incongruent
Congruent vs Intermediate
Incongruent vs Intermediate
Frequency [Hz]
Time [s]
Z-score

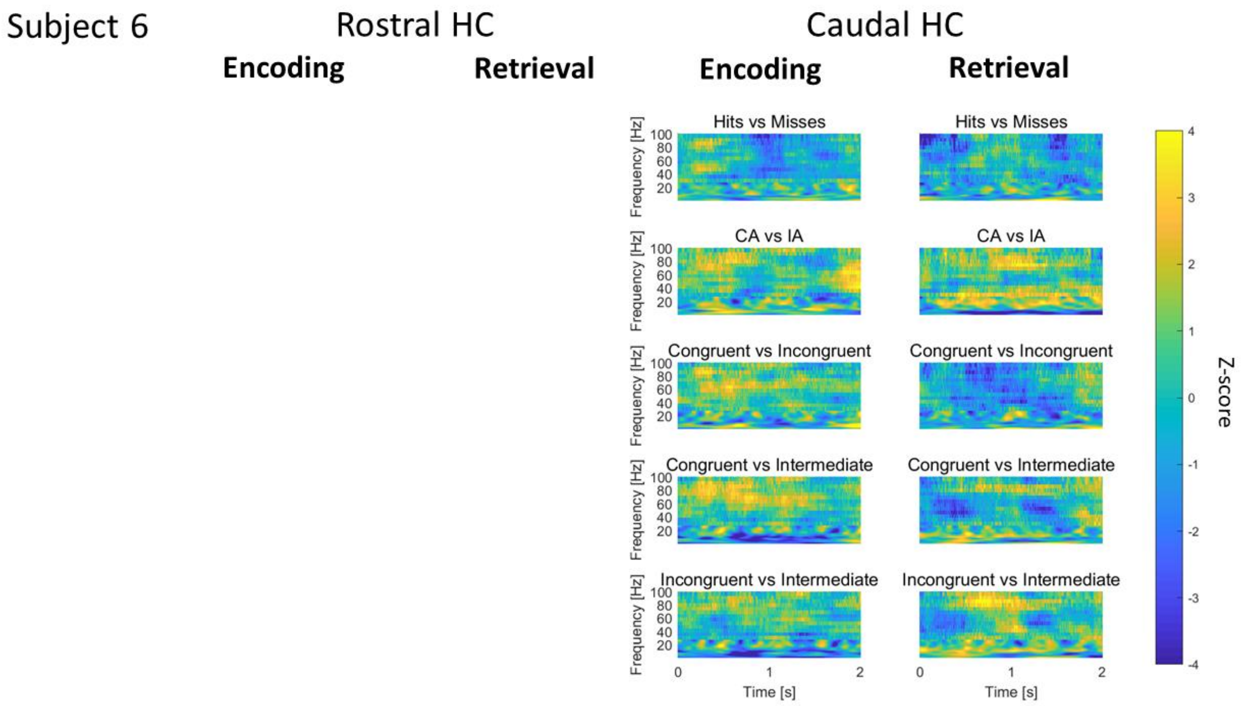

Supplementary Figures 3-8. Time-frequency maps showing memory-related and congruence-related power contrasts during encoding and retrieval in the rostral portion of the HC and in the caudal portion of the HC: results over all subjects.
